# A COVID-19 vaccination model for Aotearoa New Zealand

**DOI:** 10.1038/s41598-022-06707-5

**Published:** 2022-02-17

**Authors:** Nicholas Steyn, Michael J. Plank, Rachelle N. Binny, Shaun C. Hendy, Audrey Lustig, Kannan Ridings

**Affiliations:** 1 Te Pūnaha Matatini: the Centre for Complex Systems and Networks, Auckland, New Zealand; 2 Department of Physics, University of Auckland, Auckland, New Zealand; 3 School of Mathematics and Statistics, University of Canterbury, Christchurch, New Zealand; 4 Manaaki Whenua, Lincoln, New Zealand

**Keywords:** Infectious diseases, Applied mathematics

## Abstract

We develop a mathematical model to estimate the effect of New Zealand’s vaccine rollout on the potential spread and health impacts of COVID-19. The main purpose of this study is to provide a basis for policy advice on border restrictions and control measures in response to outbreaks that may occur during the vaccination roll-out. The model can be used to estimate the theoretical population immunity threshold, which represents a point in the vaccine rollout at which border restrictions and other controls could be removed and only small, occasional outbreaks would take place. We find that, with a basic reproduction number of 6, approximately representing the Delta variant of SARS-CoV-2, and under baseline vaccine effectiveness assumptions, reaching the population immunity threshold would require close to 100% of the total population to be vaccinated. Since this coverage is not likely to be achievable in practice, relaxing controls completely would risk serious health impacts. However, the higher vaccine coverage is, the more collective protection the population has against adverse health outcomes from COVID-19, and the easier it will become to control outbreaks. There remains considerable uncertainty in model outputs, in part because of the potential for the evolution of new variants. If new variants arise that are more transmissible or vaccine resistant, an increase in vaccine coverage will be needed to provide the same level of protection.

## Introduction

COVID-19 was first detected in Wuhan, China in late 2019^1^, before spreading globally to become a pandemic in March 2020^2^. New Zealand adopted an elimination approach early in the pandemic^3^ and, as a result, has experienced significantly lower cumulative incidence of cases and deaths than many other countries^4^. Strict border controls have been implemented to keep the virus out, including complete closure to most non-residents, and mandatory government-managed quarantine on arrival for those allowed to enter. Although this has been largely successful in preventing community transmission of COVID-19, 10 border-related re-incursions were detected in the period up to 31 March 2021^5^. However, with the development of effective vaccines, widespread vaccination could allow these border restrictions to be safely relaxed.

New Zealand’s vaccination programme began in February 2021 and almost exclusively uses the two-dose Pfizer/BioNTech BNT162b2 mRNA vaccine^6^. On 10 March 2021, the New Zealand government published their vaccine roll-out plan^7^, which identified four successive groups for vaccination: (1) 50,000 frontline border workers, managed isolation and quarantine (MIQ) staff, and their household contacts; (2) 480,000 frontline health workers and people in high-risk settings; (3) approximately 1.7 m priority individuals, primarily those in older age groups; (4) the remainder of the general population aged 16 + (approximately 2 m people). In August 2021, the age of eligibility was reduced to 12 years. As of mid December 2021, 94% of the eligible population have received at least one dose and 90% are fully vaccinated.

Quantifying the effect of the vaccine roll-out on the population-level risk of severe health outcomes and on the degree of population immunity is critical for informing the response to future border-related outbreaks and decisions about when and how to relax border restrictions. Evidence suggests the Pfizer/BioNTech BNT162b2 mRNA vaccine is highly effective against mild and severe COVID-19, with efficacy against disease from stage three clinical trials reported to be 95% (90.3%, 97.6%)^8^. Data from Israel^9^, a country with high vaccination rates, suggests an effectiveness against mild disease of 94% (87%, 98%) and an effectiveness against severe disease of 92% (75%, 100%). The same study also found high effectiveness against documented infection with SARS-CoV-2 of 92% (88%, 95%), suggesting similar effectiveness against transmission^10^.

Since late 2020, several SARS-CoV-2 variants of concern have been identified that are more transmissible and/or more resistant to vaccines^11^. The Alpha (B.1.1.7) variant, which became dominant in the UK in early 2021, is estimated to be 43–90% more transmissible than ancestral SARS-CoV-2^12^. The Delta (B.1.617.2) variant, which was first identified in India and subsequently displaced the Alpha variant in the UK to become dominant in mid 2021, has a secondary attack rate that is approximately 40% higher than Alpha^13^. The effectiveness of the Pfizer/BioNTech vaccine after 2 doses against symptomatic disease has been estimated to be around 88% for the Delta variant, compared to around 93% for Alpha^14^. This suggests that there is limited reduction in vaccine effectiveness for Delta after two doses (although the same study showed a bigger reduction in effectiveness after only one dose). Effectiveness against hospitalisation and mortality remains high against Delta, with Public Health England estimating 96% effectiveness against hospitalisation^15^.

While we do not consider specific variants in this work, we present results for three values of the basic reproduction number ${R}_{0}=3.0$, $4.5$, and $6.0$. These are broadly reflective of spread of the original variant of SARS-CoV-2, the Alpha variant, and the Delta variant respectively. However, there is uncertainty as to the exact value of ${R}_{0}$ for the Delta variant in particular, and these three values should be interpreted as a range of low, medium and high transmission scenarios. While there is considerable uncertainty in vaccine effectiveness, our baseline assumptions are chosen to best represent the current understanding of the vaccine’s effect on the Delta variant after two doses.

We develop an age-structured vaccination model that we implement in two ways: (A) a deterministic SEIR model; and (B) a stochastic branching process model. The former is intended to analyse population-level dynamics and is similar to other SEIR models developed for COVID-19^16,17^. The latter is intended to analyse small-scale outbreaks where stochasticity plays an important role and is similar to our previous branching process model for COVID-19 spread^18,19^. This builds on earlier models used to inform the response to previous outbreaks of COVID-19 in New Zealand^19,20^ by incorporating the effects of vaccination, age-structured contact rates, and contact tracing. This paper does not present new empirical data, but the model assumptions and parameter estimates are informed by previously published data from New Zealand and international studies.

The primary objective of this paper is to develop a model that can be used to understand the potential impacts of COVID-19 at various stages in the vaccine roll-out and inform a range of policy questions. The model does not yet account for heterogeneity of vaccine coverage, inequities in health outcomes for different groups^21^, or waning of immunity over time. Extensions to the model to account for these will be necessary in future work.

## Results

### Effect of vaccination on transmission potential

**Figure 1:**
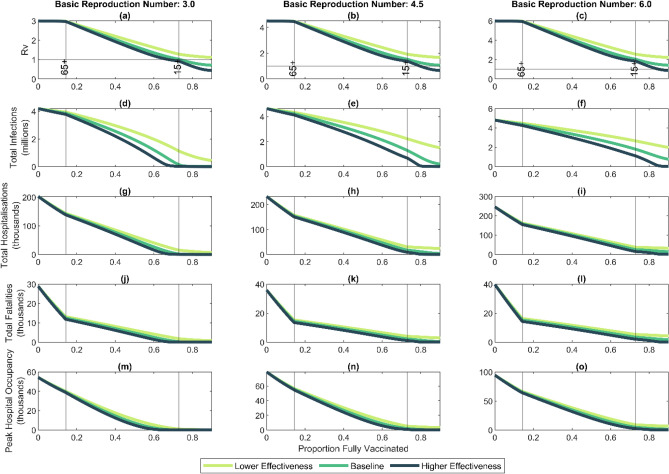
High vaccine coverage greatly reduces potential morbidity and mortality but, for a highly transmissible variant (${R}_{0}=6$), population immunity is unlikely to be achieved via vaccination alone: (**a**–**c**) effective reproduction number ${R}_{v}$ after vaccination; (**d**–**f**) total infections; (**g**–**i**) total hospitalisation; (**j**–**l**) total fatalities; (**m**–**o**) peak hospital occupancy for an unmitigated epidemic over a two-year period as a function of total vaccine courses administered, assuming there is no further vaccination after the outbreak begins. Results are shown for three values of the basic reproduction number: ${R}_{0}=3.0$, ${R}_{0}=4.5$, ${R}_{0}=6.0$. Along the horizontal axis, the roll-out begins in the 65 + year-old age group; once 90% of the 65 + year old group is vaccinated (left-hand vertical line), vaccination of the 15–64-year-old group begins; once 90% of the 15–64-year-old group is vaccinated (right-hand vertical line), vaccination of the under 15-year-old age group begins. Vaccine effectiveness assumptions as shown in Table 1.

We investigate potential outcomes of an epidemic occurring at different stages in the vaccine roll-out (Fig. 1). A key aim of national vaccination programmes is to vaccinate enough people that the effective reproduction number is below 1 without the need for other interventions. The proportion of the population that need to be vaccinated to achieve this is known as the population immunity threshold. In this section, we estimate the reproduction number ${R}_{v}$ at different stages in the vaccine roll-out and predict the vaccination coverage required to reach the population immunity threshold.

**Table 1:** Vaccine effectiveness parameters.

Effectiveness	Baseline (%)	Lower effectiveness (%)	Higher effectiveness (%)
Against infection ($${e}_{I})$$	70	50	90
Against transmission given infection ($${e}_{T})$$	50	40	50
Against disease given infection ($${e}_{D})$$	80	80	80
Implied overall transmission reduction	85	70	95
Implied overall protection against severe disease	94	90	98

These are chosen to reflect estimates of the effectiveness of the Pfizer-BioNTech mRNA vaccine after 2 doses^24^. The lower effectiveness parameters represent a potential SARS-CoV-2 variant of concern that partially evades immunity provided by the Pfizer vaccine. See Supplementary Information sec. 3 for more details. The overall reduction is transmission is given by $1-(1-{e}_{I})(1-{e}_{T})$, and the overall protection against severe disease is $1-\left(1-{e}_{I}\right)\left(1-{e}_{D}\right)$.

With an assumed basic reproduction number of $${R}_{0}=3$$ before vaccination, reaching the population immunity threshold ($${R}_{v}<1)$$ requires vaccinating 74% of the total population including children under baseline vaccine effectiveness assumptions, and 68% under the high effectiveness assumptions (Table 1, Fig. 1a). Under lower effectiveness parameters, the population immunity threshold cannot be reached. Similar results have been found for Australia^22^ and the UK^23^. With an assumed basic reproduction number of $${R}_{0}=4.5$$ before vaccination, reaching the population immunity threshold under baseline effectiveness assumptions requires vaccinating 91% of the total population (Fig. 1b). Even under the higher effectiveness assumption, vaccination of a significant proportion of under 15-year-olds is needed to reach the population immunity threshold. With an assumed basic reproduction number of $${R}_{0}=6.0$$ before vaccination, the population immunity threshold would require almost 100% vaccine coverage in the baseline scenario, and would require vaccination of under-15-year-olds even in the higher effectiveness scenario (Fig. 1c).

The first stage of the vaccine roll-out, where older age groups are vaccinated, does not substantially reduce the effective reproduction number at an overall population level (Fig. 1a–c). These age groups have lower contact rates than other age groups, so contribute a relatively small amount to overall transmission. These groups are at greatest risk of severe health outcomes, however, so vaccinating them is expected to substantially reduce the potential morbidity and mortality if a large outbreak were to occur (see below).

**Figure 2:**
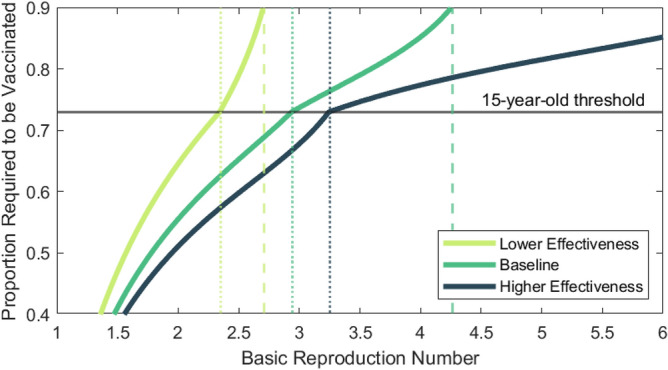
Number of vaccinated individuals required to reach the population immunity threshold (i.e. ${R}_{v}=1$) for varying values of ${R}_{0}$ under the same age-prioritised roll-out sequence and maximum 90% coverage as in this figure. Points above the thin horizontal line require vaccination of under 15-year-olds. Vertical dotted lines indicate the largest value of ${R}_{0}$ for which the population immunity threshold can be reached with 90% coverage of over 15-year-olds. Vertical dashed lines indicate the largest value of ${R}_{0}$ for which the population immunity threshold can be reached with 90% coverage of the total population.

The vaccine coverage required to reach the population immunity threshold for different values of ${R}_{0}$ and under the three vaccine effectiveness scenarios are shown in Table 2 and Fig. 2. Under the baseline vaccine effectiveness assumptions, the maximum value of the basic reproduction number for which population immunity can be achieved with 90% coverage of over 15-year-olds is ${R}_{0}=2.9$. If ${R}_{0}$ is between 3.0 and 4.3, population immunity can be achieved with 90% coverage of the whole population. If ${R}_{0}$ is above 4.3 (which is likely for the Delta variant of concern^24^), population immunity cannot be achieved under baseline vaccine assumptions without a vaccine coverage of more than 90% of the whole population.

**Table 2:** Population immunity threshold estimates for each vaccine effectiveness scenario at three different values of ${R}_{0}$.

	Baseline	Lower effectiveness	Higher effectiveness
Vaccine effectiveness	${e}_{I}=70\%, {e}_{T}=50\%$	${e}_{I}=50\%, {e}_{T}=40\%$	${e}_{I}=90\%, {e}_{T}=50\%$
${R}_{0}=3.0$	74%	95%*	68%
${R}_{0}=4.5$	91%*	–	80%
${R}_{0}=6.0$	98%*	–	85%

Estimates assume a structured roll-out, beginning in 65 + year-olds, then 15–64 year-olds, and finally under 15-year olds, with up to 90% of each group vaccinated. Estimates with an asterisk are greater than 90% and assume equal coverage in all age groups.

### Open borders scenario

To model the effect of relaxing border restrictions, which currently require 14 days of quarantine for all international arrivals from outside a safe travel zone, we assume there are five non-vaccinated imported cases per day. This is a counterfactual scenario in which no interventions are made to control the epidemic beyond vaccination. This does not mean that the number of hospitalisations and fatalities reported below would be expected to occur, but it demonstrates that, under these scenarios, a significant public health response would still be needed to prevent a major epidemic and the associated health impacts.

We run the deterministic SEIR implementation for two years (730 days) in a population with either 90% vaccination coverage across all age groups, or 90% vaccination coverage across over 15-year-olds. If the model is run for a longer time period, more cases, hospitalisations, and fatalities occur, even if ${R}_{v}<1$. The choice of five imported non-vaccinated infected individuals per day is arbitrary and, over the two-year simulation, equates to approximately 2500 symptomatic cases, 200 hospitalisations, and 35 fatalities among imported cases, independent of vaccine effectiveness and coverage, and the assumed basic reproduction number ${R}_{0}$. To focus on the effects of community transmission, we do not include imported cases in the results presented below. Other case importation rates are tested in Supplementary Information sec. 2.5.

With vaccine coverage of 90% of the over 15-year-old age groups and an assumed value of ${R}_{0}=3.0$ before vaccination, we found that ${R}_{v}=1.02$ under baseline vaccine effectiveness parameters. This is slightly above the population immunity threshold, so each imported infection leads to many local cases, and there are substantial health impacts in this scenario (Table 3). Even in the higher effectiveness scenario, ${R}_{v}=0.93$, so each imported infection still leads to multiple local cases, and the health impacts are non-negligible. This highlights the importance of increasing vaccination coverage even if population immunity is achieved.

**Table 3:** Results from an unmitigated epidemic with ${R}_{0}=3$ (upper), ${R}_{0}=4.5$ (middle), and ${R}_{0}=6$ (lower) before vaccination and 90% vaccine coverage for over 15-year-olds.

	Baseline	Lower effectiveness	Higher effectiveness
$${{\varvec{R}}}_{0}=3.0$$			
Vaccine effectiveness	${e}_{I}=70\%, {e}_{T}=50\%$	${e}_{I}=50\%, {e}_{T}=40\%$	${e}_{I}=90\%, {e}_{T}=50\%$
${R}_{v}$	1.02	1.28	0.93
Infections	150,000 (44%)	1,100,000 (61%)	25,000 (18%)
Hospitalisations	2000 (35%)	15,000 (47%)	310 (15%)
Fatalities	230 (35%)	1800 (47%)	37 (15%)
Peak in hospital	32 (after 415 days)	990 (after 210 days)	N/A
$${{\varvec{R}}}_{0}=4.5$$			
${R}_{v}$	1.53	1.92	1.39
Infections	1,300,000 (44%)	2,200,000 (58%)	690,000 (18%)
Hospitalisations	17,000 (35%)	30,000 (47%)	8,800 (15%)
Fatalities	2200 (35%)	4100 (47%)	1100 (15%)
Peak in hospital	2000 (after 140 days)	5200 (after 100 days)	750 (after 174 days)
$${{\varvec{R}}}_{0}=6.0$$			
${R}_{v}$	2.04	2.56	1.85
Infections	1,800,000 (44%)	2,700,000 (58%)	1,100,000 (19%)
Hospitalisations	25,000 (35%)	37,000 (47%)	15,000 (15%)
Fatalities	3400 (35%)	5300 (47%)	2000 (15%)
Peak in hospital	4700 (after 90 days)	8900 (after 70 days)	2400 (after 110 days)

All results are given to 2 significant figures and only include the locally acquired cases, not those imported from overseas. All scenarios assume protection against severe disease given infection of ${e}_{D}=80\%$. Numbers in parentheses are the percentage of infections/hospitalisations/fatalities that occur in vaccinated individuals.

For scenarios with higher values of ${R}_{0}$, which are more likely for the Delta variant, vaccinating 90% of over 15-year-olds is not sufficient to bring ${R}_{v}$ near or below 1, meaning that there is the potential for a self-sustaining epidemic wave to occur resulting in large numbers of hospitalisations and fatalities. Corresponding results with a vaccine coverage of 90% of the whole population including children are shown in Table 4.

**Table 4:** Results from an unmitigated epidemic with ${R}_{0}=3$ (upper), ${R}_{0}=4.5$ (middle) and ${R}_{0}=6$(lower) before vaccination and 90% vaccine coverage for the entire population (including children).

	Baseline	Lower effectiveness	Higher effectiveness
$${{\varvec{R}}}_{0}=3.0$$			
Vaccine effectiveness	${e}_{I}=70\%, {e}_{T}=50\%$	${e}_{I}=50\%, {e}_{T}=40\%$	${e}_{I}=90\%, {e}_{T}=50\%$
${R}_{v}$	0.71	1.11	0.43
Infections	13,000 (73%)	440,000 (82%)	3700 (47%)
Hospitalisations	240 (35%)	6700 (47%)	100 (15%)
Fatalities	26 (35%)	730 (47%)	11 (15%)
Peak in hospital	N/A	210 (after 310 days)	N/A
$${{\varvec{R}}}_{0}=4.5$$			
${R}_{v}$	1.06	1.67	0.65
Infections	210,000 (73%)	1,500,000 (82%)	8700 (47%)
Hospitalisations	3800 (35%)	24,000 (47%)	240 (15%)
Fatalities	410 (35%)	2900 (47%)	26 (15%)
Peak in hospital	N/A	3200 (after 120 days)	N/A
$${{\varvec{R}}}_{0}=6.0$$			
${R}_{v}$	1.41	2.22	0.87
Infections	770,000 (73%)	2,000,000 (82%)	26,000 (47%)
Hospitalisations	14,000 (35%)	32,000 (47%)	710 (15%)
Fatalities	1700 (35%)	4300 (47%)	77 (15%)
Peak in hospital	1300 (after 150 days)	6600 (after 80 days)	N/A

All results given to 2 significant figures and do not include imported cases, only the resulting community transmission cases. All scenarios assume protection against severe disease given infection of ${e}_{D}=80\%$. Numbers in parentheses are the percentage of infections/hospitalisations/fatalities that occur in vaccinated individuals.

In all scenarios, a significant proportion of infections, hospitalisations and deaths occur in vaccinated individuals, which is the expected outcome when vaccine coverage is high and effectiveness is less than 100%. This demonstrates the need for continued testing and other public health measures to be directed at vaccinated as well as non-vaccinated individuals.

Figure 1d–o show the number of infections, hospitalisations and fatalities in an unmitigated epidemic at different stages in the vaccine roll-out sequence. Although vaccinating older age groups first does not greatly reduce in the total number of infections, it does lead to a sharper reduction in hospitalisations and fatalities. These scenarios assume that the vaccination programme ceases once the outbreak begins and so they represent the potential effect of opening the border once a certain number of individuals are fully vaccinated.

### Control of border-related outbreaks

**Figure 3:**
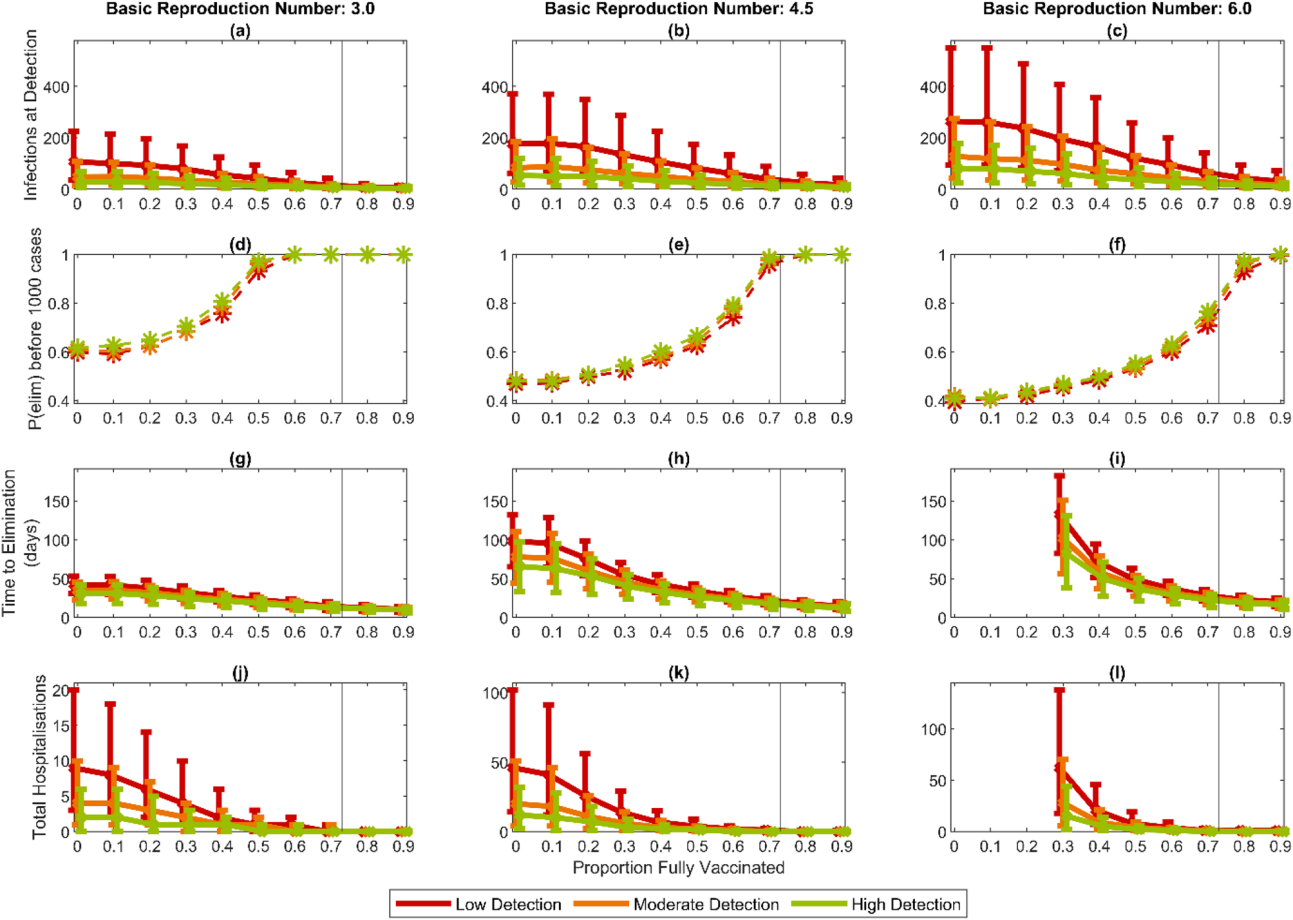
Detection and control of small outbreaks as vaccine coverage increases: (**a**–**c**) number of infections when a community outbreak is first detected; (**d**–**f**) probability an outbreak is eliminated before reaching 1000 cases without population-level interventions; (**g**–**i**) time from detection to elimination with population-level interventions; (**j**–**l**) total number of hospitalisations before outbreak is eliminated. Results are shown for three values of ${R}_{0}$ and three values of the probability of testing for symptomatic individuals in the period before the outbreak is detected: ${p}_{detect}^{pre}=5\%$ (red), ${p}_{detect}^{pre}=10\%$ (amber) and ${p}_{detect}^{pre}=15\%$ (green). In (**d**–**f**), case isolation and contact tracing are introduced after outbreak detection giving 43% reduction in ${R}_{eff}$; in (**g**–**l**), population-level restrictions are also introduced after outbreak detection giving a total 81% reduction in ${R}_{eff};$ note that in (**i**) and (**l**) when ${R}_{0}=6$, an 81% reduction in ${R}_{eff}$ is insufficient to achieve elimination when vaccine coverage is less than around 20%. The black vertical line represents the point in the vaccine roll-out at which vaccination of under 15-year-olds begins. Points are the median and error bars represent the interquartile range of 10,000 independently initialised realisations of the stochastic model.

In this section, we use the stochastic branching process implementation of the model to simulate outbreaks seeded by a single infected case. The results from these simulations can be interpreted as the expected number of community cases per fully infectious case arriving at the border. We consider four outputs: the number of infections when an outbreak is first detected; the probability that an outbreak eliminates before it reaches 1000 cumulative infections, without population-level interventions; the time from detection until elimination, defined as 14 days since the last infection event, with fixed post-detection interventions; and the number of hospitalisations that occur before elimination is achieved (Fig. 3). As these outbreaks are all relatively small we do not model fatalities. In all simulations, case isolation and contact tracing measures are introduced when the outbreak is first detected, resulting in a 44% reduction in the effective reproduction number (see “Methods”). In simulations of the time to elimination, additional population-wide interventions are implemented when the outbreak is first detected, modelled by an additional reduction of 67% in the effective reproduction number. This is consistent with estimates of the relative reduction in transmission from control measures in response to previous outbreaks in New Zealand and may be achieved via Alert Level changes or other public health interventions.

Until high levels of vaccination are achieved, probability of detection is the most important factor affecting the size of outbreaks at detection (Fig. 3a–c). This shows the ongoing importance of high rates of community testing for early outbreak detection. In a completely non-vaccinated population, the probability of elimination is around 60% when ${R}_{0}=3.0$, around 50% when ${R}_{0}=4.5$, and around 40% when ${R}_{0}=6$. (Fig. 3d–f). This is partly due to stochasticity of transmission, meaning that a relatively high proportion of infected individuals do not transmit the virus. Vaccination of the older age groups at the start of the roll-out does not substantially change the probability of elimination. However, once the roll-out in those under 65 years old begins (> 16% fully vaccinated), the probability of elimination steadily increases with vaccine coverage. Once ${R}_{v}$ drops below approximately $1.77$, case isolation and contact tracing are theoretically sufficient to bring the effective reproduction number below 1. This occurs when approximately 47%, 66% or 78% of the population have been vaccinated when ${R}_{0}=3$, $4.5$ or $6$ respectively. Once coverage is greater than these values, assuming contact tracing and case isolation remain effective even in very large outbreaks, all outbreaks will eventually eliminate. In practice, however, the observed probability of elimination in Fig. 3d–f may still be less than 1 because some outbreaks still reach 1,000 cases before elimination.

As the detection probability or vaccination coverage increases, outbreaks can be eliminated more rapidly (Fig. 3g–i). It may be possible to lift population-level restrictions before elimination is formally achieved, but we do not model this here. The number of hospitalisations decreases sharply with vaccination coverage (Fig. 3j–l), thanks to protection of high-risk groups in the early stages of the vaccine roll-out. When ${R}_{0}=6$, however, the assumed reduction of 81% in the reproduction number due to post-detection interventions is not sufficient for outbreak control at low vaccine coverage (Fig. 3i), suggesting that stronger measures may be required. However, this result should be viewed with caution because we currently lack data on transmission of new variants under stay-at-home restrictions or other strong interventions. The assumption we make that a given control measure leads to the same proportional reduction in transmission regardless of ${R}_{0}$ may not always be valid^25^.

## Discussion

We have investigated an age-structured model for transmission of COVID-19 in a partially vaccinated population, parameterised to represent New Zealand’s age-structure and age-specific contact rates. In New Zealand the Pfizer/BioNTech vaccine was originally approved for use in people aged over 16 years but has since also been approved for 12–15-year-olds. Our results show that, under baseline vaccine effectiveness assumptions and with ${R}_{0}=4.5$, vaccination of over 90% of the population will likely be necessary to reach the population immunity threshold, defined as a reproduction number that is less than 1 in the absence of other interventions. This would require vaccination of under 16 year olds. If ${R}_{0}=6$ (which could represent a highly transmissible SARS-CoV-2 variant such as Delta^13^), population immunity would require approximately 98% of the total population, something which is unlikely to be achievable in practice.

The model also assumes that vaccine coverage is evenly spread throughout the population within each age group. In reality, we expect coverage to be heterogeneous. This means that, even if ${R}_{v}<1$ at a national level, communities with relatively low vaccine coverage (or relatively high contact rates) could still have ${R}_{v}>1$ locally, which would expose them to major outbreaks. It will be important to pay attention to vaccine coverage in different geographic and socioeconomic groups to identify at-risk communities and prioritise them for vaccination.

Our results show that vaccination of the priority age group (over 65 years) at the beginning of the programme does not greatly reduce the potential for community transmission of the virus, but sharply reduces hospitalisations and fatalities by protecting the highest-risk groups in the model. However, there are other risk factors we have not accounted for here. Māori and Pacific peoples have previously been shown to be at greater risk of hospitalisation^21^ and fatality^26^ after accounting for age and reported comorbidities. Any strategy to minimise negative health outcomes from COVID-19 should prioritise these groups for vaccination. It will also be essential to ensure that Māori and Pacific communities can access the vaccine in a way that meets their cultural, linguistic and social needs^27^. This includes removing costs and barriers, and ensuring that it is easy and convenient to access the vaccine. Once high-risk groups are vaccinated, extending high coverage levels to lower-risk groups including young people and, provided regulatory approval is obtained, children will be crucial to minimising the potential for transmission and reaching the population immunity threshold.

Whether or not New Zealand reaches the theoretical population immunity threshold, the higher vaccination coverage is, the more collective protection the population has against infections, hospitalisations and deaths from COVID-19. Conversely, even if population immunity is achieved, transmission can still occur. As a rule-of-thumb, for any effective reproduction number less than 1, the average number of cases caused by a single re-introduction is given by ${R}_{eff}/(1-{R}_{eff})$^28^. For example, if ${R}_{eff} = 0.95$, there will be an average of 19 community cases for every border re-introduction. This means that, regardless of the population immunity threshold, it will be essential to continue to vaccinate as many people as possible to minimise the combined potential for transmission and health impacts.

Nguyen et al.^17^ also investigated alternative age-based vaccine prioritisation schemes for New Zealand and focused on the minimum vaccine allocation required to reach the population immunity threshold. The results from our SEIR model are comparable to those of Nguyen et al.^17^ when ${R}_{0}=4.5$ and we investigated in greater detail the outcomes for a more transmissible variant with ${R}_{0}=6$. In addition, our stochastic model allowed us to investigate the effect of increasing levels of vaccine coverage on the dynamics and control of small border-related incursions of COVID-19. Our results also show that, until a high proportion of the population is vaccinated, the probability of case detection is still an important determinant of outbreak size. This demonstrates that high rates of community testing for surveillance purposes will remain crucial throughout the vaccine roll-out to minimise the risk of a large outbreak. Although the model ignores seasonal effects, these will be particularly important during the winter influenza season when the incidence of symptoms consistent with COVID-19 is relatively high and seasonality may increase the transmission potential of SARS-CoV-2^29^.

As vaccine coverage increases, the stringency and duration of population-level restrictions (e.g. via New Zealand’s COVID-19 Alert Level system) required to eliminate an outbreak decreases. Under the baseline scenario with ${R}_{0}=4.5$, once approximately 60% of the population is fully vaccinated, the model estimates that it will be possible to eliminate any new outbreaks with case isolation and contact tracing alone. However, it is extremely unlikely that the contact tracing system would perform at the level required if there were a large number of imported cases that triggered multiple concurrent outbreaks, which was the situation New Zealand faced in March 2020. In the medium term, a modest reduction in transmission could be achieved by baseline measures such as mask use, widespread community testing, and isolation of symptomatic individuals. For example, if 70% of symptomatic cases are isolated (with 80% effectiveness) an average of 2 days after onset of symptoms, ${R}_{eff}$ is reduced by 11%. This would imply that effective population immunity could be achieved when vaccination reduces ${R}_{eff}$ to approximately $1.1$. However, this will still require very high vaccine coverage and is subject to the caveats discussed above about low coverage and high contact groups remaining vulnerable.

Together these results suggest that, until we get close to the population immunity threshold, a major public health response that included significant interventions would still be required to control any resurgent outbreaks and prevent a major epidemic. Nonetheless, as vaccination coverage increases, it may be possible for the requirement for 14 day government-managed isolation period to be replaced with a combination of testing, shorter quarantine periods or home isolation for arrivals from low-risk countries. Future work will model the risk from different border policies and different traveller risk profiles. However, strong border restrictions designed to keep COVID-19 out of the community are likely to be required until very high vaccination coverage is achieved nationally.

We have investigated outcomes when the overall vaccine effectiveness against transmission is 70%, 85% and 95%. Published evidence shows the Pfizer-BioNTech mRNA vaccine is highly effective in preventing documented infection after two doses^9,30,31^, thereby implying high effectiveness against transmission^10^. Despite this, there is still some uncertainty around the exact effectiveness of this vaccine in reducing transmission. Furthermore, the ongoing emergence of variants of concern that are potentially more transmissible^12^ or less responsive to vaccines^32^ could mean that, in future, ${R}_{0}$ is substantially higher or vaccine effectiveness is substantially lower than the values assumed in this paper^33^. Our baseline vaccine effectiveness parameters are also consistent with more recent estimates for the Delta variant^34^, while our lower effectiveness scenario represents a variant of concern that is able to partially evade immunity, such as the Beta variant^35^. Higher values of ${R}_{0}$ or lower values of vaccine effectiveness against transmission mean that higher vaccination coverage is required to obtain the same level of protection at a population level. Although the Pfizer vaccine has high effectiveness against the Delta variant after two doses, effectiveness after only one dose is significantly lower^13^. This means that administering the full course of two doses is essential to providing full immunity. Booster doses are now being rolled out^36^ and these may be required to reach the higher effectiveness scenario, or even the baseline scenario. Continued research into the pathogenicity, transmission characteristics, and responsiveness to vaccines of emerging variants of SARS-CoV-2 is needed to evaluate their effect on population immunity levels and the risk they pose.

The model contains a number of simplifications, limitations, and sources of uncertainty. Firstly, it models transmission of SARS-CoV-2 at a national level and ignores geographic and socioeconomic heterogeneities that will affect transmission rates. It also does not consider differential transmission rates in specific settings (such as households, schools and workplaces). Secondly, there is still uncertainty about the intrinsic transmissibility and vaccine effectiveness, particularly for new and potential future variants of concern. Data is still being gathered and analysed on this internationally; model results will need to be updated as new data becomes available. Thirdly, the rates of contact between age groups are based on 2008 survey data from EU countries^37^, mapped onto the current age structure of New Zealand's population. We have also assumed an age-dependent susceptibility profile based on modelled estimates^38^, which are subject to uncertainty and are difficult to measure directly. If actual age-dependent contact rates or susceptibility have a significantly different structure than assumed, this would affect how cases are distributed across age groups, which would change model outputs for hospitalisations and deaths. Fourthly, the results describe model outcomes under specific assumptions about the interventions taken in response to cases. These are either counterfactuals modelling a completely unmitigated epidemic, or elimination scenarios that assume strong and effective interventions are implemented for as long as is needed to eliminate an outbreak. We do not attempt to predict the outcomes of other control responses or behavioural factors that may change the effectiveness of interventions such as contact tracing, quarantine or prolonged restrictions. Finally, the model also ignores the effects of seasonality, waning immunity, population dynamics (births, deaths, ageing and migration) and environmental factors (e.g. pollution, latitude, humidity, and temperature) which have been shown to be correlated with SARS-CoV-2 transmission rates or severity^29,39–41^. These factors will introduce additional challenges in achieving and maintaining high levels of population immunity. In particular, there is growing evidence that immunity against infection from the Pfizer vaccine wanes over a period of several months, but high levels of immunity can be provided by a booster dose^36^. Models can be used to investigate the consequences of waning immunity and booster doses^42^ and the effects of this in New Zealand will be addressed in future work.

We have focused on hospitalisations as a metric of demand on the healthcare system and have not modelled other outcomes, such as ICU occupancy, which may be equally important. A more detailed clinical pathways model could be applied to our model outputs, for example using an age-specific profile of ICU admission risk and length of ICU stay distribution. However, we note that ICU admission risk is subject to more uncertainty because of smaller numbers and significant variation between countries in observed ICU rates so we have not attempted to do this for New Zealand here.

Until the vaccine rollout is complete, retaining the elimination strategy has two key benefits. Firstly, it protects people who have not yet been offered the vaccine and the minority of people for whom the vaccine is less effective, or who cannot be vaccinated for medical reasons. Secondly, by keeping cases to a minimum, it maximises the ability of the contact tracing system to reduce transmission. This decreases the likelihood of needing to use the alert level system to control future outbreaks. Nonetheless this work provides insight into some of the opportunities to change policy settings that may arise as New Zealand’s vaccination programme rolls out.

## Methods

Full details of the SEIR and branching process models are provided in Supplementary Information sec. 1 and Supplementary Figs. S1 and S2.

### Age-structured contact rates

We used an age-structured model of SARS-CoV-2 transmission in New Zealand with the population divided into 15 five-year age bands, plus an over-75-year-old age band. The relative contact rates within and between age groups are defined by a matrix $\widehat{{\varvec{C}}}$, Prem, et al.^37^ (see Supplementary Information sec. 1.3 and Supplementary Figs. S3 and S4). Susceptibility to infection and the proportion of infections (in non-vaccinated individuals) that result in clinical disease, hospitalisation, and fatality vary by age (see Supplementary Table S1). We assume that subclinical individuals have a transmission rate that is $\tau =50\%$ of that of clinical cases^38^.

### Vaccination coverage

In each model scenario, the proportion of age group $i$ that is vaccinated, denoted ${v}_{i}$, is assumed to be fixed, i.e. we do not attempt to model simultaneous vaccination and transmission dynamics. The interplay between these dynamics is important in countries rolling out vaccination programmes at the same time as dealing with high rates of infection, but is less important for countries such as New Zealand where COVID-19 is currently eliminated. For simplicity, we assume that all individuals are either not vaccinated, or fully vaccinated (i.e. more than 7 days after their second dose). To model different staging points in the vaccine roll-out, we assume that at most 90% of any age group can be vaccinated, with vaccinations beginning in the 65 + year age groups, then in the 15–64 years age groups, and finally in the 0–14 years age groups. At a given stage in the roll-out, we assume the vaccine coverage ${v}_{i}$ is the same for each five-year age group $i$ within the same priority age band. See Supplementary Information sec. 2.2 for a more fine-grained age prioritisation.

### Vaccine effectiveness

The effectiveness of the vaccine is described by three parameters: effectiveness against infection ($${e}_{I})$$, effectiveness against transmission conditional on breakthrough infection ($${e}_{T}$$), and effectiveness against severe disease conditional on breakthrough infection ($${e}_{D})$$ (see Table 1). For simplicity, we do not model any additional reduction in mild disease beyond that provided by prevention of infection. We also assume that the vaccine is equally effective in all age groups and we do not consider the effect of any waning of immunity over time. We assume the vaccine blocks infection in an “all-or-nothing” fashion. This means that the vaccine prevents any infection from occurring in a fixed proportion $${e}_{I}$$ of the vaccinated population, with the remaining vaccinated population being fully susceptible (see Supplementary Information sec. 2.3 for an alternative assumption).

### Next generation matrix

The next generation matrix, ${NGM}_{i,j}$, defines the average number of individuals in group $i$ that will be infected by a single infectious individual in group $j$ over their whole infectious period given a fully susceptible population:

$$NG{M}_{i,j}=U{u}_{i}{t}_{I}\widehat{{C}_{j,i}}[{p}_{j}^{clin}+\tau (1-{p}_{j}^{clin})]$$

where $\widehat{{\varvec{C}}}$ is the contact matrix, ${t}_{I}$ is the mean duration of the infectious period in days, $U{u}_{i}$ describes the probability that a contact by an individual in age group $i$ with an infectious individual results in transmission, ${p}_{j}^{clin}$ is the fraction of infections in age group $j$ that are clinical, and $\tau$ is the relative infectiousness of subclinical individuals. The basic reproduction number of the age-structured model is the dominant eigenvalue of the next generation matrix, denoted ${R}_{0}=\rho (NGM)$. In model simulations, the value of the constant $U$ is chosen to give the desired value of ${R}_{0}$. When a proportion ${v}_{i}$ of age group $i$ are vaccinated (but with no immunity from prior infection), the next generation matrix $NG{M}^{v}$ is given by:

$$NG{M}_{i,j}^{v}=\left(1-{e}_{I}{v}_{i}\right)(1-{e}_{T}{q}_{j})NG{M}_{i,j}$$

where ${q}_{j}=\left(1-{e}_{I}\right){v}_{j}/(1-{e}_{I}{v}_{j})$ is the proportion of infections in age group $j$ that are vaccinated. This reflects the assumption that transmission from vaccinated individuals is reduced by ${e}_{T}$ and infection of vaccinated individuals is reduced by ${e}_{I}$. The vaccinated reproduction number is ${R}_{v}=\rho (NG{M}^{v})$.

### Initial conditions

Seed cases representing international arrivals are assumed to be non-vaccinated and clinical, are distributed over all age groups in proportion to the size of those groups, spend the full infectious period in the community, and have the same contact patterns as the general population. If some proportion of seed cases are vaccinated or subclinical, this is equivalent to a smaller number of unvaccinated, clinical seed cases; model results are not highly sensitive to this assumption (see Supplementary Fig. S11). We do not model higher vaccination or surveillance rates for border workers, who are more likely to be a seed case.

### Testing and contact tracing

We simulate the outbreak dynamics under three different levels of community testing: low, moderate, and high, corresponding to the probability of detecting a symptomatic COVID-19 case of 5%, 10%, and 15% respectively during the period of time before the outbreak is first detected. We assume there is a delay from symptom onset to the return of a positive test result of 4 days on average and that the detection rate is the same for vaccinated and non-vaccinated individuals and across all age groups. Once an outbreak is detected, we assume that testing rates increase and case isolation and contact tracing begins. As a simplified representation of this, we assume that all infections have a probability of being detected by contact tracing of 70%, with a mean delay of 6 days after the infection time. We assume that, independently of contact tracing, the probability of detecting a clinical infection as a result of testing following symptom onset increases to 40%, representing increased awareness of infection risk. Once an infection is detected, the individual is assumed to be immediately isolated so that there is no further transmission. Because the stochastic implementation of the model is used to represent relatively small outbreaks, we assume that the testing and contact tracing parameters are independent of the number of cases (i.e. we do not model a decrease in system performance as cases stretch case management capacity). Together, case isolation and contact tracing measures result in a reduction in the reproduction number of around 44% (see Supplementary Information sec. 1.2), which is consistent with estimates from empirical data from New Zealand’s March–April 2020 outbreak^20^. The individual values of the testing probabilities and delay times are less important than their combined effect on the reproduction number.

### Sensitivity analysis

Additional sensitivity analysis is shown in Supplementary Figs. S5, S6, S7, S8, S9, S10, S11, S12, S13 and S14.

### Research methods statement

All methods were carried out in accordance with relevant guidelines and regulations.

## Supplementary Information

Supplementary Information 1.

Supplementary Information 2.

## Data Availability

No new datasets are presented in this research. Code to reproduce the results is available as electronic online material and at https://github.com/nicsteyn2/TPM-COVID19-VaccineModel.
