# Supplementary material for: A COVID-19 vaccination model for Aotearoa New Zealand
Source: Sci Rep. 2022 Feb 17;12:2720. doi: 10.1038/s41598-022-06707-5 (PMC8854696; doi:10.1038/s41598-022-06707-5)
Supplement: Supplementary file 1 — Supplementary Information 1. [file 41598_2022_6707_MOESM1_ESM.docx]

# A COVID-19 Vaccination Model for Aotearoa New Zealand

# Supplementary Information

Contents

[1. Model specification 2](#_Toc90307322)

[1.1 Deterministic SEIR implementation 2](#_Toc90307323)

[1.2 Stochastic branching process implementation 10](#_Toc90307324)

[1.3 Contact matrix 12](#_Toc90307325)

[2. Sensitivity analysis 15](#_Toc90307326)

[2.1 Sensitivity to contact matrix assumptions 15](#_Toc90307327)

[2.2 Sensitivity to vaccine roll-out sequence 20](#_Toc90307328)

[2.3 Sensitivity to vaccine infection-blocking assumptions 23](#_Toc90307329)

[2.4 Approval for 12+ year-olds only 25](#_Toc90307330)

[2.5 Deterministic SEIR sensitivity to other epidemiological parameters 27](#_Toc90307331)

[2.6 Effect of testing and case isolation on reproduction number 27](#_Toc90307332)

[2.7 Stochastic branching process sensitivity to vaccine effectiveness 32](#_Toc90307333)

[2.8 Stochastic branching process sensitivity to other epidemiological parameters 33](#_Toc90307334)

[3. Effectiveness of the Pfizer-BioNTech vaccine 35](#_Toc90307335)

[3.1 Effectiveness against SARS-CoV-2 infection 35](#_Toc90307336)

[3.2 Effectiveness against transmission given breakthrough infection 37](#_Toc90307337)

[3.3 Effectiveness against symptomatic COVID-19 37](#_Toc90307338)

[3.4 Effectiveness against severe disease/hospitalisation 37](#_Toc90307339)

[3.5 Effectiveness against death 37](#_Toc90307340)

# 1. Model specification

We envisage two distinct applications for a COVID-19 vaccination model in New Zealand. The first is to consider the effects of the vaccination programme on the degree of population immunity and potential for spread in the wider community. The second is to consider the effect of vaccination on relatively small community outbreaks seeded by border cases, like those that occurred in August 2020 and February 2021 [1].

To accomplish both goals, we propose two implementations of this model: (1) a deterministic SEIR model, and (2) a stochastic branching process. The former focusses on average behaviour, allowing it to efficiently model population-level dynamics. The latter considers individual cases and stochasticity in transmission (e.g. superspreading), elements which are critical for analysing small clusters. Matlab code for both implementations of the model is provided as Electronic Supplementary Material.

## 1.1 Deterministic SEIR implementation

#### Transmission model

The SEIR model consists of five non-vaccinated compartments for each age group $i$: susceptible ($S_{i}$), exposed ($E_{i})$, clinical infectious ($I_{i})$, subclinical infectious ($A_{i})$, and recovered ($R_{i})$. The model also includes the same five compartments for vaccinated age groups, denoted with a superscript $v$. Finally, an additional compartment for vaccinated infection-immune individuals $Imm_{i}$ is included. (Figure S1, Equations S1 and S2).

The model is initialised with a proportion, $v_{i}$, of each age group vaccinated. Of these, $\left( 1-e_{I} \right)v_{i}$ are assigned to the susceptible vaccinated compartment $S_{i}^{v}$, and the remaining $e_{I}v_{i}$ are assigned to the immune vaccinated compartment $Imm_{i}$. That is, we are assuming the infection blocking aspect of the vaccine acts in an “all-or-nothing” fashion (see Sec. 2.3 for details).


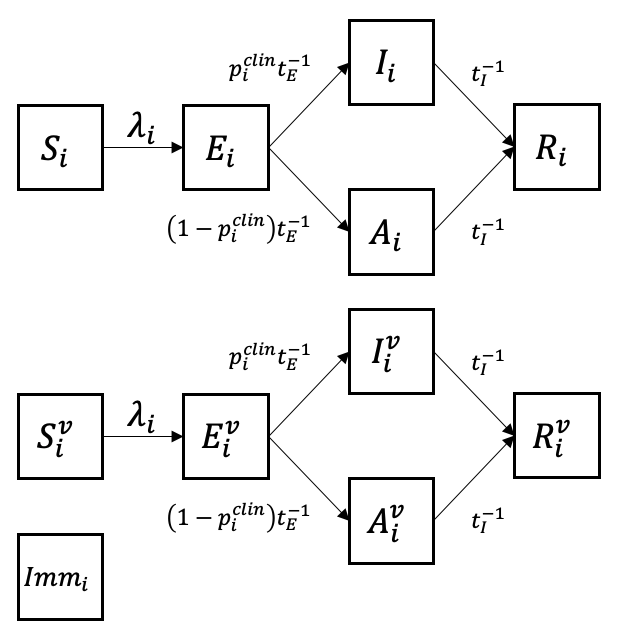


**Figure S1.** Structure diagram of the deterministic SEIR implementation. $S_{i},E_{i},I_{i},A_{i}, R_{i}$ represent the number of susceptible, exposed, clinical infectious, subclinical infectious and recovered individuals respectively in age group $i$. Superscript $v$’s indicate vaccinated compartments and $Imm_{i}$ consists of individuals that are vaccinated and immune to infection.

The model consists of 10 ordinary differential equations for each age group which are outlined in equations (S1).

| $\frac{dS_{i}}{dt}= -\lambda_{i}S_{i}$ | $\frac{dS_{i}^{v}}{dt}= -\lambda_{i}S_{i}^{v}$ |
| --- | --- |
| $\frac{dE_{i}}{dt}=\lambda_{i}S_{i}-t_{E}^{-1}E_{i}$ | $\frac{dE_{i}^{v}}{dt}=\lambda_{i}S_{i}^{v}-t_{E}^{-1}E_{i}^{v}$ |
| $\frac{dI_{i}}{dt}={p_{i}^{clin}t}_{E}^{-1}E_{i}-t_{I}^{-1}I_{i}$ | $\frac{dI_{i}^{v}}{dt}={p_{i}^{clin}t}_{E}^{-1}E_{i}^{v}-t_{I}^{-1}I_{i}^{v}$ |
| $\frac{dA_{i}}{dt}=\left( 1-p_{i}^{clin} \right)t_{E}^{-1}E_{i}-t_{I}^{-1}A_{i}$ | $\frac{dA_{i}^{v}}{dt}=\left( 1-p_{i}^{clin} \right)t_{E}^{-1}E_{i}^{v}-t_{I}^{-1}A_{i}^{v}$ |
| $\frac{dR_{i}}{dt}=t_{I}^{-1}(I_{i}+A_{i})$ | $\frac{dR_{i}^{v}}{dt}=t_{I}^{-1}(I_{i}^{v}+A_{i}^{v})$ |

**Equations (S1).** Equations are identical for the vaccinated and non-vaccinated groups. The model is typically solved with initial conditions $S_{i}(0)=N_{i}(1-v_{i})$, $S_{i}^{v}(0)=N_{i}v_{i}(1-e_{I})$, and $Imm_{i}=N_{i}v_{i}e_{I}$ (although $Imm_{i}$ is fixed so does not feature in these equations).

In equations (S1), $t_{E}$ and $t_{I}$ are the mean time spent in the exposed compartment (i.e. the latent period) and mean time spent in the infectious compartment respectively. As is standard in epidemiological compartment models, this implicitly assumes that the length of time spent in each compartment is exponentially distributed [2]. More complex distributions such as Erlang distributions can be modelled by introducing additional compartments though this comes at the expense of additional model complexity and does not typically have a major effect on total epidemic size. We assume that the mean infectious period $t_{I}$ is the same for clinical and subclinical infections. This is a model simplification and ignores the potential effect of self-isolation in symptomatic individuals. Note that the stochastic branching process model (see Section 1.2 below) includes a more realistic generation time distribution and explicitly models the effect of symptomatic testing and case isolation. In addition, sensitivity analysis (see Section 2.5 below) investigates the effect of varying the parameter $t_{I}.$

The proportion of infections in age group $i$ that are clinical is given by $p_{i}^{clin}$, and $\lambda_{i}$ is the infection force acting on age group $i$, defined by

$$\begin{aligned} \lambda_{i}=U\frac{u_{i}}{N_{i}}\sum_{j} \left( I_{j}+\tau A_{j}+\left( 1-e_{T} \right)\left( I_{j}^{v}+\tau A_{j}^{v} \right)+t_{I}m_{j} \right)C_{j,i} \#\left( S2 \right) \end{aligned}$$

In Eq. (S2), $N_{i}$ is the total number of individuals in age group $i,$ $u_{i}$ is the relative susceptibility to infection of age group $i$, and $U$ is a constant chosen so the model is run with the desired value of $R_{0}$. Together $Uu_{i}$ can be thought of as the probability of an individuals in age group $i$ becoming infected given contact with an “average” infectious individual. The number of imported cases per day in age group $j$ is given by $m_{j}$ – these are assumed to be clinical, not vaccinated, and spend their entire infectious period in the country. If some proportion of seed cases are vaccinated or subclinical, this produces an infection force that is equivalent to a smaller number of unvaccinated, clinical seed cases. $\tau$ is the relative infectiousness of subclinical individuals. These cases are not assigned to a compartment so are not counted towards total cases, hospitalisations, and fatalities.

The average infectious period, $t_{I}$, is assumed to be 5 days [3]. While the cited paper uses a latent period of 3 days, we use $t_{E}=2.55$ days for consistency with the time scale of the branching process implementation. Sensitivity analysis performed on $t_{E}$ shows it only affects the timing of epidemic peaks, rather than the final size or health outcomes (see Section 2.5 below).

*Disease, hospitalisation and fatality rates*

The proportion of infections (in non-vaccinated individuals) that result in clinical disease, hospitalisation, and fatality vary by age and are taken from international literature [4,5] – see Table S1. This data is presented in 10-year age groups. We use linear interpolation matched to the mid-points of each age group to derive parameters in 5-year age groups. For the 75+ year age group, for symptomatic disease rates we use the reported symptomatic fraction for the 70+ year age group (the oldest group reported in [5]), and for hospitalisations and fatalities, a weighted average of the rates reported for the 75-79 year and the 80+ year age groups in [4].

The relative transmission rate $\tau$ of subclinical infections is important for modelling contact tracing and case isolation as clinical infections are more likely to receive a test than subclinical infections. We assume that the fraction of infections that are clinical increases with age. A consequence of these assumptions is that infections in older age groups are more infectious on average than those in younger groups, although the difference is relatively small (see Supplementary Information sec 2).

*Age-dependent susceptibility and infectiousness*

There is evidence that susceptibility to infection varies by age [6]. Multiple studies suggest that young people (typically considered to be under 20 years old, although different studies have used different age bands) may be 50% less susceptible than adults [7-9]. We include this in the transmission model using an age-structured relative susceptibility term estimated by Davies, et al. [7] – see Table S1.

There is less certainty around age-dependent infectiousness, with some studies suggesting younger individuals are more infectious [9], while others suggesting they are less infectious [8]. In our model, the youngest age group have an effective transmission rate that is 14% lower than the oldest age group as a consequence of the assumption that subclinical infections are more common in younger individuals and are less infectious than clinical infections.

*Vaccine effectiveness*

Multiple studies have found that the effectiveness of the Pfizer-BioNTech BNT162b2 mRNA vaccine against documented infection to be of the order 90% [10-12], although there is early evidence of reduced effectiveness in older individuals (64% (14%, 84%) in long-term care facility residents in Denmark) [13] and the Beta (B.1.351) variant of concern (75% (70.5%, 78.9%) in Qatar) [14]. Furthermore, it is unclear whether effectiveness against documented infection arises solely from infection prevention, or is due in part to lower infectiousness or shorter time windows of infectiousness. In either case, effectiveness against documented infection is expected to be a lower bound on overall effectiveness against transmission [15]. There is further evidence of transmission reduction in breakthrough infections, with an odds ratio of infection for household contacts of a vaccinated index case relative to an non-vaccinated index case estimated to be approximately 0.5 [16]. Based on the evidence available, we use effectiveness against infection of $e_{I}=70\%$ and effectiveness against transmission given breakthrough infection of $e_{T}=50\%$ as baseline assumptions. This is equivalent to an overall reduction in transmission of $1-\left( 1-e_{I} \right)\left( 1-e_{T} \right)=85\%$. These parameters are similar to those used in for the Delta variant in recent models of the UK government roadmap for relaxation of restrictions [17].

Efficacy against all disease from clinical trials has been found to be 95% (90.3%, 96.7%) [18] with similar effectiveness against severe disease [14]. For the baseline assumption, we set the effectiveness against severe disease given breakthrough infection to be $e_{D}= 80\%$, which gives an overall effectiveness against severe disease of $1-\left( 1-e_{I} \right)\left( 1-e_{D} \right)=94\%$. We assume that the vaccine effectiveness against fatality is the same as the effectiveness against severe disease.

To provide results over a plausible range for vaccine effectiveness, we consider two additional scenarios, one with lower effectiveness parameters, $e_{I}=50\%$ and $e_{T}=40\%$ (which gives a 70% reduction in transmission), and one with higher effectiveness parameters, $e_{I}=90\%$ and $e_{T}=50\%$ (which gives a 95% reduction in transmission). Although a 70% reduction in transmission is lower than published studies indicate, we include this scenario because there is evidence that existing vaccines are less effective against some SARS-CoV-2 variants of concern (e.g. the Beta variant) [19] and there is significant uncertainty about how effectiveness will change for future potential variants. In all cases $e_{D}$ is set to 80%, implying overall effectiveness against severe disease of 90% and 98% in the lower and higher effectiveness scenarios respectively. Vaccine effectiveness parameters are shown in Table 1 of the Main Text.

*Hospitalisation and fatality submodel*

The number of hospitalisations and fatalities are also tracked through a hospitalisation subroutine. This consists of an additional three compartments: $H_{i}$, $F_{i}$, and $Disch_{i}$. These represent individuals in hospital, fatalities, and those discharged from hospital. The cumulative number of hospitalisations is given by the sum in $F_{i}$ and $Disch_{i}$. The same three compartments are repeated for vaccinated individuals. (Figure S2, Equations S3).


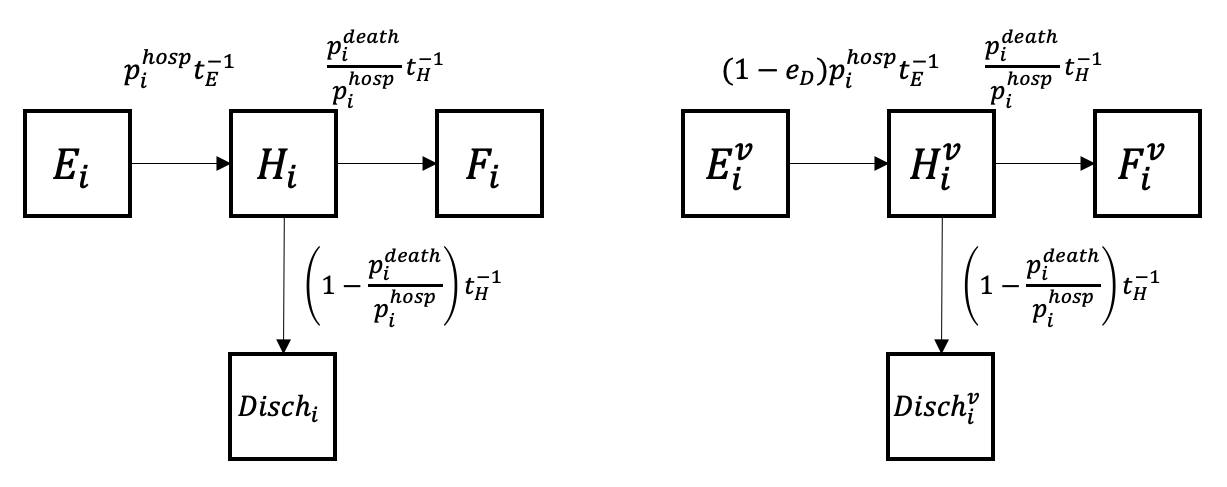


**Figure S2.** Structure diagram of the hospitalisation and fatality subroutine. $E_{i}$, $H_{i}, Disch_{i},$and $F_{i}$ represent the number of exposed individuals (from the primary transmission model), number of current hospitalisations, number of discharged, and number of fatalities respectively.

$$\frac{dH_{i}}{dt}=p_{i}^{hosp}t_{E}^{-1}E_{i}-t_{H}^{-1}H_{i}$$

$$\frac{dF_{i}}{dt}=\frac{p_{i}^{death}}{p_{i}^{hosp}}t_{H}^{-1}H_{i}$$

$$\frac{dDisch_{i}}{dt}=\left( 1-\frac{p_{i}^{death}}{p_{i}^{hosp}} \right)t_{H}^{-1}H_{i}$$

$$\frac{dH_{i}^{v}}{dt}=\left( 1-e_{D} \right)p_{i}^{hosp}t_{E}^{-1}E_{i}^{v}-t_{H}^{-1}H_{i}^{v}$$

$$\frac{dF_{i}^{v}}{dt}=\frac{p_{i}^{death}}{p_{i}^{hosp}}t_{H}^{-1}H_{i}^{v}$$

$$\frac{dDisch_{i}^{v}}{dt}=\left( 1-\frac{p_{i}^{death}}{p_{i}^{hosp}} \right)t_{H}^{-1}H_{i}^{v}$$

**Equations (S3).**

In these equations, $p_{i}^{hosp}$ and $p_{i}^{death}$ are the probability of being hospitalised and dying conditional on being infected. This formulation assumes that the length of time spent in the hospitalised compartment is exponentially distributed with mean $t_{H}$. Other distributions of length of hospital stay could be modelled. For example, including a series of $n$ hospitalised compartments, each with mean length of stay $t_{H}/n$ results an on overall length of stay that is Erlang distributed with mean $t_{H}$ and shape parameter $n$. However, this assumption does not affect the transmission dynamics or the total number of hospitalisations, only the peak number of people hospitalised at any one time and results are not sensitive to this assumption. For simplicity, we assume that exposed individuals are hospitalised with the same average delay as they become infectious, and that hospitalised individuals die with the same average delay as they are discharged. This only has a minor effect on the timing of the peak hospital occupancy estimates.

| **Parameter** | **Branching implementation** | **SEIR implementation** | | **Source** |
| --- | --- | --- | --- | --- |
| Basic reproduction number $R_{0}$ | 3.0, 4.5, 6.0 | | |  |
| Relative infectiousness of subclinical individuals ($\tau)$ | 50% | | | [7] |
| Latent period $t_{E}$ | N/A | 2.55 days | | Suppl. 1 |
| Infectious period $t_{I}$ | N/A | 5 days | | [3] |
| Generation time distribution $T_{G}$ | Weibull(2.83, 5.67)*  *(mean = 5.05 days)* | N/A | | [20] |
| Average hospital length of stay $t_{H}$ | 8 days | | | [21] |
| Probability of detecting a case $p_{detect}$ | Scenario dependent | | N/A |  |
| Incubation period $T_{ons}$ | Gamma(5.8, 0.95)*  *(mean = 5.51 days)* | | N/A | [22] |
| Delay from infection to contact tracing $T_{trace}$ | Exponential(6 days) | | N/A | Assumed |
| Delay from symptom onset to detection $T_{detect}$ | Exponential(4 days) | | N/A | Assumed |
| Age groups | [0-4, 5-9, 10-14, 15-19, 20-24, 25-29, 30-34, 35-39, 40-44, 45-49, 50-54, 55-59, 60-64, 65-69, 70-74, 75+] | | |  |
| Population size $N$ | N/A | 5 million | |  |
| Population distribution $N_{i}/N$ | N/A | [5.98%, 6.39%, 6.56%, 6.17%, 6.59%, 7.40%, 7.44%, 6.62%, 6.08%, 6.41%, 6.43%, 6.38%, 5.77%, 4.90%, 4.24%, 6.64%] | | [23] |
| Proportion of infections causing clinical disease by age group $p_{clin,i}$ | [0.544, 0.555, 0.577, 0.5985, 0.6195, 0.6395, 0.6585, 0.6770, 0.6950, 0.7118, 0.7273, 0.7418, 0.7553, 0.768, 0.78, 0.8008] | | | [5] |
| Proportion of infections resulting in hospitalisation by age group $p_{hosp,i}$ | [0, 0.0001, 0.0003, 0.0029, 0.0079, 0.0164, 0.0283, 0.0364, 0.0405, 0.0523, 0.0718, 0.0907, 0.1089, 0.13, 0.154, 0.178] | | | [4] |
| Proportions of infections resulting in fatality by age group $p_{death,i}$ | [0, 0.00003, 0.00006, 0.0001, 0.0002, 0.0004, 0.0007, 0.001, 0.0014, 0.0027, 0.0049, 0.0093, 0.016, 0.0252, 0.0369, 0.0664] | | | [4] |
| Relative susceptibility by age group (compared to 60-64 year-olds) $u_{i}$ | [0.462, 0.457, 0.445, 0.558, 0.795, 0.934, 0.974, 0.977. 0.942, 0.931, 0.942, 0.965, 1.00, 0.977, 0.896, 0.856] | | | [7] |

**Table S1.** Table of parameter values. *Weibull and gamma distributions are reported with shape parameter then scale parameter in parenthesis.

## 1.2 Stochastic branching process implementation

Evidence suggests overdispersion in the distribution of the number of secondary cases in SARS-CoV-2 transmission: a few individuals typically account for a large amount of transmission (“superspreading”) [24]. To include this in the branching process, each clinical infected individual $l$ is assigned a reproduction number $R_{l}^{clin}$ given by:

$$\begin{aligned} R_{l}^{clin}={Ut_{I}Y}_{l}\left( 1-V_{l}e_{T} \right)\sum_{j} {u_{j}C}_{a_{l},j} \end{aligned}$$

where $Y_{l}$ is drawn independently from a gamma distribution with shape $k$ and scale $1/k$, $a_{l}$ is the age group of individual $l$, and $V_{l}$ is an indicator variable that equals 1 when individual $l$ is vaccinated and 0 otherwise. If the individual is subclinical then $R_{l}^{sub}=\tau R_{l}^{clin}$. Under this formulation, the total number of people infected by a randomly selected clinical individual has a negative binomial distribution with dispersion parameter $k$. Here we set $k=0.5$, which is within the range of values estimated for SARS-CoV-2 transmission in New Zealand [25] and other countries [26].

The number of secondary cases that would be generated by individual $l$ at timestep $t$ in a non-vaccinated population is then given by:

$$\begin{aligned} S_{l}^{t}\sim Poisson\left( F^{ctrl}\left( t \right)F_{l}^{isol}\left( t \right)R_{l}\int_{t}^{t+\Delta t} w\left( \tau-t_{inf,l} \right)d\tau\right) \end{aligned}$$

Assuming $F^{ctrl\left( t \right)}=F_{l}^{isol\left( t \right)}=1$, the distribution of $\sum_{t} S_{l}^{t}$ is negative binomial with mean $R_{l}$ and overdispersion $k=0.5$. $F^{ctrl}\left( t \right)$ represents the reduction in transmission as a result of any population-level control measures at time $t$ and is equal to 1 if there are no control measures in place. $F_{l}^{isol}(t)$ represents the reduction in transmission as a result of case isolation and contact tracing and is equal to 1 before individual $l$ has been isolated, and equal to 0 after. $t_{inf,l}$ is the time that individual $l$ was infected, and $w\left( t \right)$ is the probability density function for the generation time distribution, which is assumed to be a Weibull distribution with mean 5 days and standard deviation 1.9 days [20].

Each would-be secondary infection is randomly assigned an age group, with probability of being assigned to age group $j$ of $\frac{u_{j}C_{a_{l},j}}{\sum_{j} u_{j}C_{a_{l},j}}$, and is then assigned to the vaccinated class with probability $v_{j}$ and to the clinical class with probability $p_{j}^{clin}$. Would-be secondary infections in the vaccinated class have probability $e_{I}$ of being prevented (i.e. not infected). Clinical individuals have onset dates drawn from a gamma distribution with mean 5.51 days and standard deviation 2.29 days [20]. Clinical non-vaccinated individuals are assigned to the hospitalised class with probability $p_{j}^{hosp}{/p}_{j}^{clin}$, and similarly clinical vaccinated individuals are assigned to the hospitalised class with probability $\left( 1-e_{D} \right)p_{j}^{hosp}/p_{j}^{clin}$. Finally, hospitalised individuals are assigned to the fatality class with probability $p_{j}^{died}/p_{j}^{hosp}$.

The branching process is simulated in time steps of $\Delta t=1 day$.

*Case detection, isolation and controls*

A simple case detection and contact tracing model is also implemented. Before an outbreak is detected, symptomatic individuals are assumed to have a probability $p_{detect}^{pre}$ of getting a test and being detected and subsequently isolated. The time from symptom onset to detection is drawn from an exponential distribution with mean $t_{detect}$ and we assume individuals are immediately isolated on detection. We assume there is no testing of asymptomatic individuals in the period before an outbreak is detected.

Once an outbreak is detected, contact tracing begins and all existing and future infections are assumed to be detected by contract tracing with probability $p_{trace}$. For simplicity, we assume this probability is the same for symptomatic and asymptomatic individuals and independent of other cases. Traced individuals are isolated with an exponentially distributed delay with mean $t_{trace}$ from the time of infection (or from the time the outbreak was first detected if this was later than the time of infection). Non-traced clinical cases are also detected with probability $p_{detect}^{post}$ and isolated with mean delay of $t_{detect}$ days from symptom onset. This models symptom-triggered testing for individuals that are missed by contact tracing. Typically we assume $p_{detect}^{post}>p_{detect}^{pre}$ to model greater symptom awareness and higher testing rates once an outbreak is detected. There is no testing of untraced asymptomatic individuals.

The effect of case isolation and contact tracing on the effective reproduction number $R_{eff}$ after outbreak detection in the model can be calculated analytically. Individuals that are contact traced (with probability $p_{trace})$ spend an average of $P(T_{G}<T_{trace})$ of their infectious period in the community prior to isolation, where $T_{G}$ is a random variable representing the generation time. The probability that a randomly chosen individual in age group $j$ is not traced but is detected through symptom-triggered testing is $\left( 1-p_{trace} \right)p_{detect}^{post}p_{j}^{clin}$ and they spend an average of $P(T_{G}<T_{onset}+T_{detect})$ of their infectious period in the community. As the branching process is simulated in time steps of 1 day, the random variable representing the generation time $T_{G}$ is the ceiling of the Weibull random variable defined in Table S1.

This implies the elements of the post-detection next generation matrix are:

$$\begin{aligned} NGM_{i,j}^{trace}=\alpha_{j}NGM_{i.j} \#\left( S4 \right) \end{aligned}$$

where:

$$\alpha_{j}=1-p_{trace}\left( 1-p_{detect}^{post}p_{j}^{clin} \right)P\left( T_{G}>T_{trace} \right)-\left( 1-p_{trace} \right)p_{detect}^{post}p_{j}^{clin}P\left( T_{G}>T_{ons}+T_{detect} \right)-p_{trace}p_{detect}^{post}p_{j}^{clin}P\left( T_{G}>\min\left( T_{trace}, T_{ons}+T_{detect} \right) \right)$$

and the implied reduction in $R_{eff}$ from case isolation and contact tracing is:

$$1-\frac{\rho\left( NGM^{trace} \right)}{\rho\left( NGM \right)}$$

Baseline parameter values for testing and tracing are $p_{trace}=0.7, t_{trace}=6 days, p_{detect}^{post}=0.4, t_{detect}=4 days$. The individual choices for the parameter values are less important than their combined effect on $R_{eff}$. For these values, $P\left( T_{G}>T_{trace} \right)=0.583$, $P\left( T_{G}>T_{ons}+ T_{detect} \right)=0.206$, and $P\left( T_{G}>\min\left( T_{trace}, T_{ons}+T_{detect} \right) \right)=0.648$, leading to a reduction in$R_{eff}$ from contact tracing of 43.7% in a non-vaccinated population.

Under current assumptions, aside from a very small effect due to age-structured clinical rates, the effectiveness of contact tracing and case isolation (measured by a percentage reduction in $R_{eff}$ without contact tracing) is largely invariant to vaccination levels. Future work will be required to consider how vaccination may change this. In such a case it may be useful to define different probabilities of detection and tracing for vaccinated and non-vaccinated individuals.

## 1.3 Contact matrix

In the absence of New Zealand specific contact data, we use a synthetic contact matrix $C$ by Prem, et al. [27] for both model implementations. This matrix is constructed from international POLYMOD data [28], fit to the New Zealand age, household, and work structures. The elements of this matrix $C_{i,j}$ give the average daily number of contacts that an individual in group $i$ has with individuals in group $j$. This implies that the total number of daily contacts that occur between an individual in group $i$ and an individual in group $j$ is $N_{i}C_{i,j}$ where $N_{i}$ is the size of group $i$. Therefore by symmetry, the contact matrix should satisfy the detailed balance condition $N_{i}C_{i,j}=N_{j}C_{j,i}$ but it does not satisfy this condition, which has implications on its use in modelling.

The contact matrices by Prem et al and POLYMOD [27,28] have been used in various modelling studies. Some define the SEIR infection pressure on age group $i$ as $\lambda_{i}\propto\sum_{j} C_{j,i}I_{j}$ [28,29], whereas others define the infection pressure as $\lambda_{i}\propto\sum_{j} \frac{C_{i,j}I_{j}}{N_{j}}$ [27,30,31]. If the contact matrix does not satisfy the detailed balance condition, these give different results.

It is not obvious which method is more correct, so we impose detailed balance by using the modified contact matrix defined by:

$$\begin{aligned} \hat{C_{i,j}}=\frac{1}{2}\left( C_{i,j}+\frac{N_{j}}{N_{i}}C_{j,i} \right) \end{aligned}$$

This ensures both expressions of the infection pressure produce identical results and can be thought of as “averaging” over both methods. This contact matrix is shown in Figure S3. We test the implications of making different assumptions about the contact matrix in Supplementary Information section 2.1.


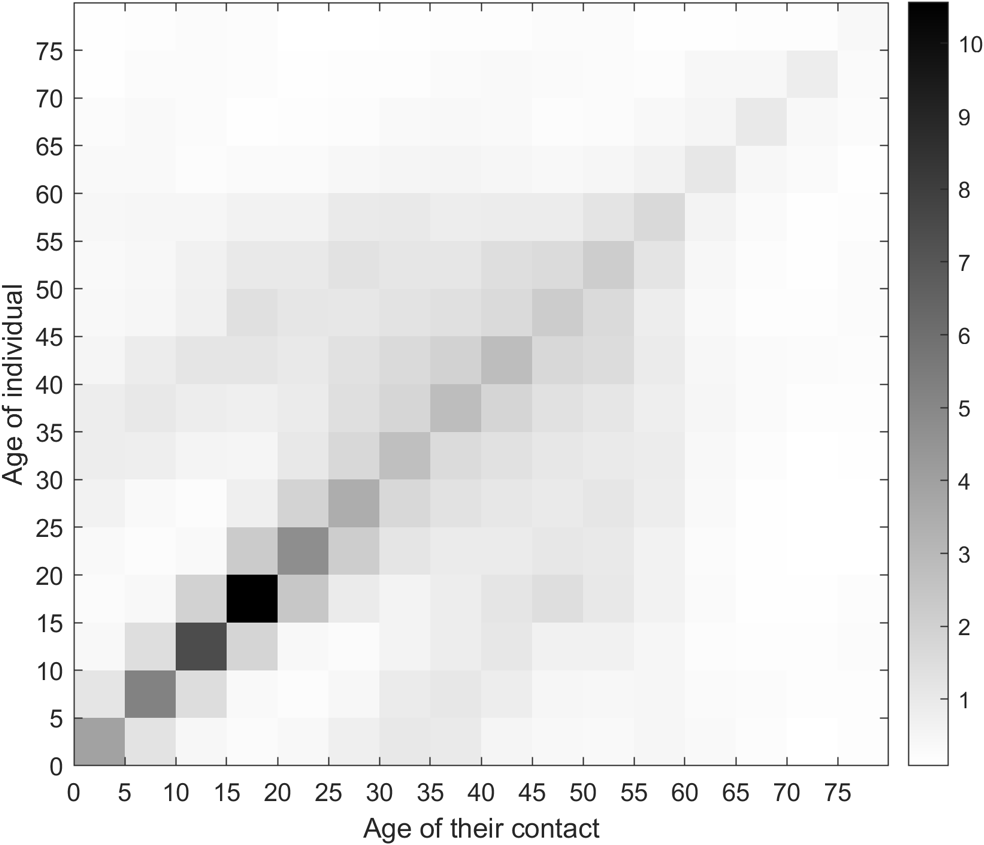


**Figure S3.** Visualisation of the modified contact matrix derived from [27] by imposing a symmetry condition described in the text. Darker colours indicate more contacts. The shading of the $(i,j)$ pixel represents the mean number of contacts an individual in age group $i$ has with individuals in age group $j$.

# 2. Sensitivity analysis

## 2.1 Sensitivity to contact matrix assumptions

We begin by considering six next-generation-matrices:

$$\begin{aligned} NGM_{i,j}\propto\frac{1}{2}\left( C_{j,i}+\frac{N_{i}}{N_{j}}C_{i,j} \right)u_{i}[p_{j}^{clin}+\tau(1-p_{j}^{clin})]\#\left( S5 \right) \end{aligned}$$

$$\begin{aligned} NGM_{i,j}\propto C_{j,i} u_{i}[p_{j}^{clin}+\tau(1-p_{j}^{clin})]\#\left( S6 \right) \end{aligned}$$

$$\begin{aligned} NGM_{i,j}\propto\frac{N_{i}}{N_{j}}C_{i,j}u_{i} [p_{j}^{clin}+\tau(1-p_{j}^{clin})]\#\left( S7 \right) \end{aligned}$$

$$\begin{aligned} NGM_{i,j}\propto\frac{1}{2}\left( C_{j,i}+\frac{N_{i}}{N_{j}}C_{i,j} \right)\#\left( S8 \right) \end{aligned}$$

$$\begin{aligned} NGM_{i,j}\propto N_{i} [p_{j}^{clin}+\tau(1-p_{j}^{clin})]\#\left( S9 \right) \end{aligned}$$

$$\begin{aligned} NGM_{i,j}\propto\frac{1}{2}\left( C_{j,i}+\frac{N_{i}}{N_{j}}C_{i,j} \right)\left[ p_{j}^{clin}+\tau\left( 1-p_{j}^{clin} \right) \right]\#(S10) \end{aligned}$$

The first four are based on the Prem, et al. [27] contact matrix $C$. Our implementation is given in Eq. (S5), which can be thought of as an “average” of Eq. (S6) and Eq. (S7) that ensures the detailed balance condition holds. Eq. (S6) uses the matrix in the same way as [28,29] while Eq. (S7) uses the matrix in the same way as [27,31]. Eq. (S8) assumes that clinical and subclinical individuals have the same infectiousness. Eq. (S9) assumes proportional mixing, where individuals interact with other age groups proportional to their size. Finally, Eq. (S10) reproduces our implementation of Eq. (S5) without age-based susceptibility.

The dominant eigenvectors of these next-generation-matrices give the pseudo-steady age distribution of infections assuming a fully susceptible population (Figure S4). They can be thought of as representing the expected age distribution of cases before any significant immunity has accumulated.

Critically our implementation in Eq. (S5) places slightly more weighting on older age groups than Eq. (S6) and slightly less than Eq. (S7). Thus, with respect to negative health outcomes, it is more pessimistic than Eq. (S6) but more optimistic than Eq. (S7). The similarity between Eq. (S5) and (S8) suggests the assumption that subclinical individuals are less infectious does not significantly alter the transmission dynamics.

Proportional mixing in Eq. (S9) assumes significantly higher contact between older age groups and the rest of the population, hence health outcomes from models that use this are expected to be significantly worse. Finally, removing age-based susceptibility in Eq. (S10) results in under-20-year-olds, who typically have more contacts, contributing to a large amount of spread.


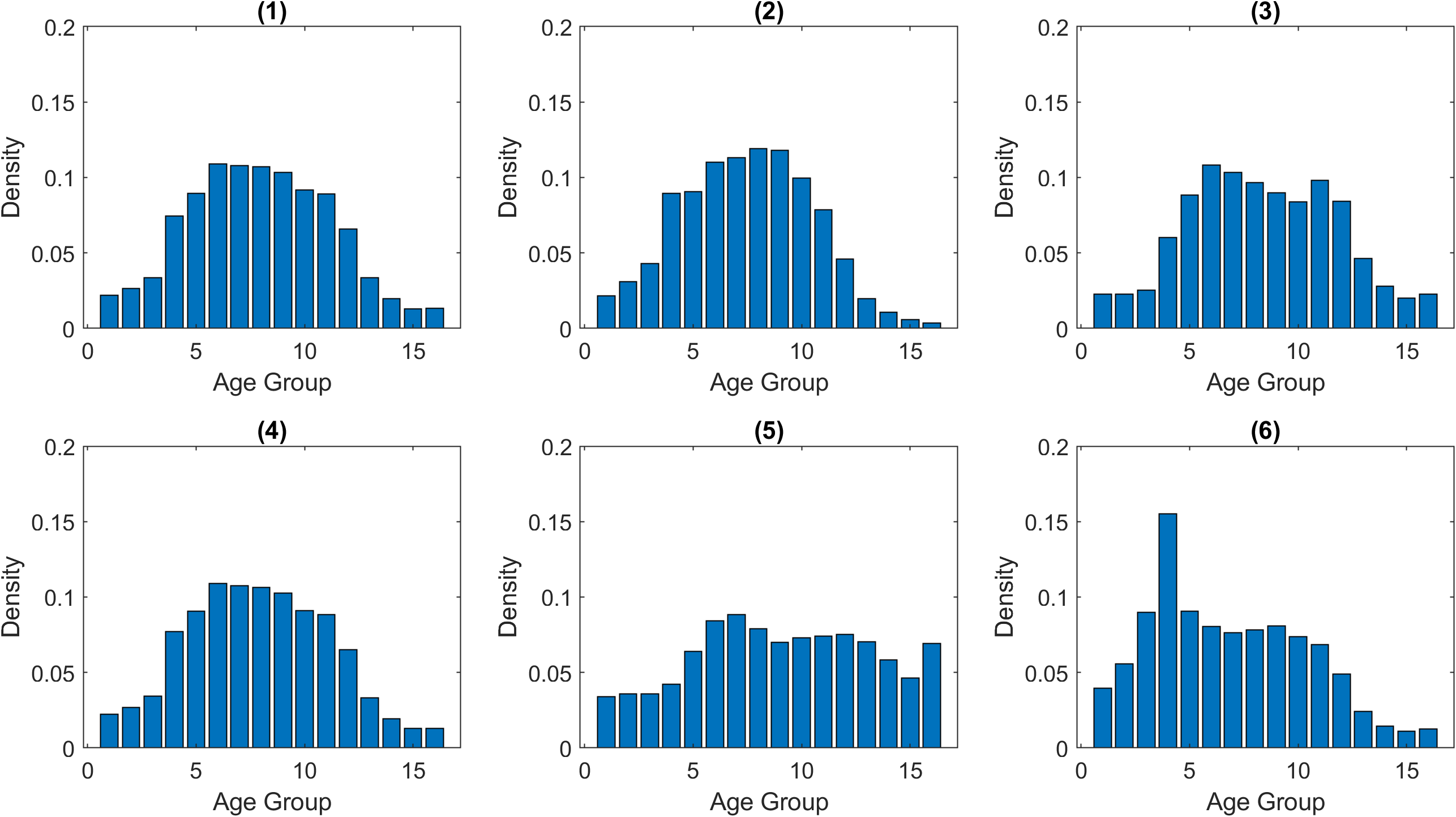


**Figure S4.** Dominant eigenvector of the six next-generation-matrices defined in equations (S5)-(S10) above.

Varying the relative importance of age groups on transmission has implications for the effect of vaccination on the reproduction number. We reproduce the results from Figure 1a-c of the Main Text under the same vaccine roll-out assumptions and baseline parameters for three of the contact matrices described above: Eq. (S5) standard, Eq. (S9) proportional mixing, and Eq. (S10) no age based susceptibility (Figure S5). Since the relative importance of children in Eq. (S10) is increased, in scenarios like this where older individuals are vaccinated first, more vaccinations are required to reach the population immunity threshold.

Assuming baseline vaccine effectiveness, we reproduce the results from Tables 3 and 4 in the Main Text under three contact assumptions: the standard model, proportional mixing, and no age based susceptibility (Tables S2 and S3).


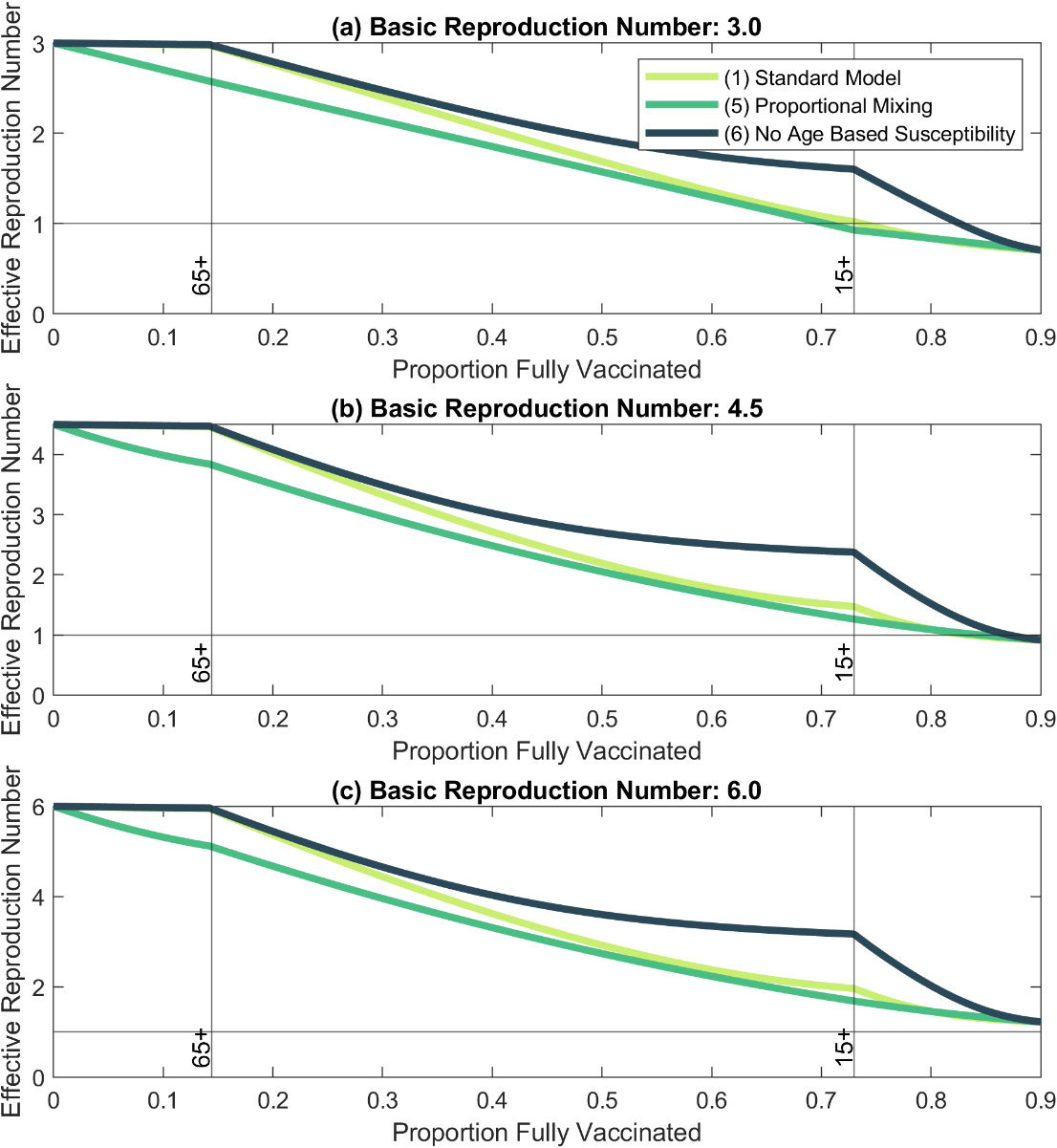


**Figure S5.** Vaccinated reproduction number under three different contact assumptions: the standard model, proportional mixing, and no age based susceptibility. Baseline vaccine effectiveness is assumed for all scenarios.

| $\boldsymbol{R}_{\boldsymbol{0}}\boldsymbol{=3.0}$ | **Standard** | **Proportional mixing** | **No age-based susceptibility** |
| --- | --- | --- | --- |
| $R_{v}$ | 1.02 | 0.93 | 1.60 |
| Infections | 150,000 (44%) | 60,000 (55%) | 990,000 (31%) |
| Hospitalisations | 2,000 (35%) | 1,400 (35%) | 8,900 (35%) |
| Fatalities | 230 (35%) | 240 (35%) | 1,100 (35%) |
| Peak in hospital | 32 (after 420 days) | N/A | 1000 (after 140 days) |
| $\boldsymbol{R}_{\boldsymbol{0}}\boldsymbol{=4.5}$ | **Standard** | **Proportional mixing** | **No age-based susceptibility** |
| $R_{v}$ | 1.53 | 1.39 | 2.40 |
| Infections | 1,300,000 (43%) | 1,200,000 (53%) | 1,600,000 (36%) |
| Hospitalisations | 17,000 (35%) | 25,000 (35%) | 18,000 (35%) |
| Fatalities | 2,200 (35%) | 4,400 (35%) | 2,400 (35%) |
| Peak in hospital | 2,000 (after 140 days) | 2,300 (after 160 days) | 3,500 (after 80 days) |
| $\boldsymbol{R}_{\boldsymbol{0}}\boldsymbol{=6.0}$ | **Standard** | **Proportional mixing** | **No age-based susceptibility** |
| $R_{v}$ | 2.04 | 1.86 | 3.20 |
| Infections | 1,800,000 (44%) | 1,800,000 (50%) | 1,900,000 (39%) |
| Hospitalisations | 25,000 (35%) | 35,000 (35%) | 24,000 (35%) |
| Fatalities | 3,400 (35%) | 6,100 (35%) | 3,300 (35%) |
| Peak in hospital | 4,700 (after 90 days) | 6,000 (after 100 days) | 5,900 (after 60 days) |

**Table S2.** Results from an unmitigated epidemic with 90% vaccine coverage of the over 15-year-old age groups, under three different contact matrices (standard contact matrix Eq. (S5), proportional mixing Eq. (S9), no age-based susceptibility Eq. (S10)). Values in parenthesis give percentage of infections/hospitalisations/fatalities that occur in vaccinated individuals.

| $\boldsymbol{R}_{\boldsymbol{0}}\boldsymbol{=3.0}$ | **Standard** | **Proportional mixing** | **No age-based susceptibility** |
| --- | --- | --- | --- |
| $R_{v}$ | 0.71 | 0.71 | 0.71 |
| Infections | 13,000 (73%) | 16,000 (73%) | 13,000 (73%) |
| Hospitalisations | 240 (35%) | 420 (35%) | 380 (35%) |
| Fatalities | 26 (35%) | 75 (35%) | 21 (35%) |
| $\boldsymbol{R}_{\boldsymbol{0}}\boldsymbol{=4.5}$ | **Standard** | **Proportional mixing** | **No age-based susceptibility** |
| $R_{v}$ | 1.06 | 1.06 | 1.06 |
| Infections | 210,000 (73%) | 260,000 (73%) | 190,000 (73%) |
| Hospitalisations | 3,800 (35%) | 6,900 (35%) | 2,800 (35%) |
| Fatalities | 410 (35%) | 1,200 (35%) | 310 (35%) |
| Peak in hospital | 85 (after 310 days) | 150 (after 310 days) | 62 (after 310 days) |
| $\boldsymbol{R}_{\boldsymbol{0}}\boldsymbol{=6.0}$ | **Standard** | **Proportional mixing** | **No age-based susceptibility** |
| $R_{v}$ | 1.41 | 1.41 | 1.41 |
| Infections | 770,000 (73%) | 920,000 (73%) | 760,000 (73%) |
| Hospitalisations | 14,000 (35%) | 24,000 (35%) | 12,000 (35%) |
| Fatalities | 1,700 (35%) | 4,200 (35%) | 1,400 (35%) |
| Peak in hospital | 1,300 (after 150 days) | 2,200 (after 150 days) | 1,100 (after 150 days) |

**Table S3.** Results from an unmitigated epidemic with 90% vaccine coverage of the total population, under three different contact matrices (standard contact matrix Eq. (S5), proportional mixing Eq. (S9), no age-based susceptibility Eq. (S10)). Values in parenthesis give percentage of infections/hospitalisations/fatalities that occur in vaccinated individuals.

## 2.2 Sensitivity to vaccine roll-out sequence

In the main paper we assume that the population is separated into three groups: 65+ year-olds, 15-64-year-olds, and under-15-year-olds. The New Zealand government recently announced that the under 65 year old age group will be prioritised in 10-year age bands, although there is no end-date for the vaccination of a given age band and there is likely to be significant overlap between vaccination of different age bands. Here, we investigate the effects of a more fine-grained age-structured vaccine roll-out, starting in 65+ year-olds before progressing through the rest of the population in 5-year age bands, with the same assumed 90% maximum coverage in any one group.

The results (Figures S6, S7, S8 and S9) show that a more age-structured roll-out results in a slower decrease in the reproduction number, but a faster reduction in the expected health impacts of any outbreak. The more structured roll-out implies a slower reduction in $R_{v}$ due to vaccination. This occurs as groups with higher contact are vaccinated later in the roll-out. However, the health implications of any widespread outbreak decrease faster under this assumption, as older individuals that are more at risk are vaccinated earlier in the roll-out.


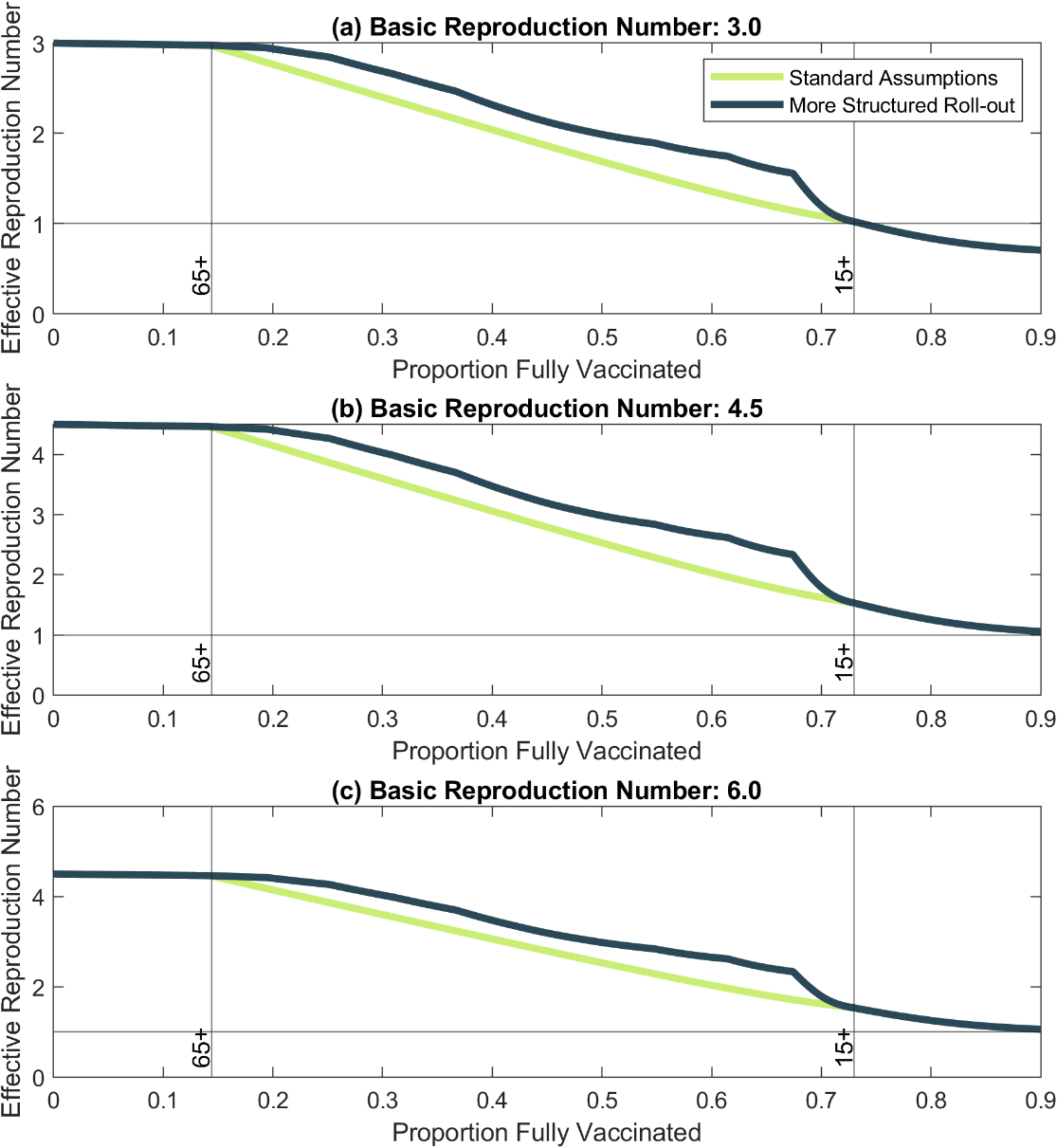


**Figure S6.** Effective reproduction number $R_{v}$ after vaccination as a function of total vaccine courses administered, with a maximum of 90% coverage in any age group, for (a) $R_{0}=3$, (b) $R_{0}=4.5$, and (c) $R_{0}=6.0$. Default vaccine effectiveness parameters are used ($e_{I}=70\%$, $e_{T}=50\%$). Standard assumptions represent the roll-out described in the Main Text, with the 15-64 year old age bands being grouped together for vaccination. The more structured scenario assumes vaccination of this group occurs in successive 5-year groups.


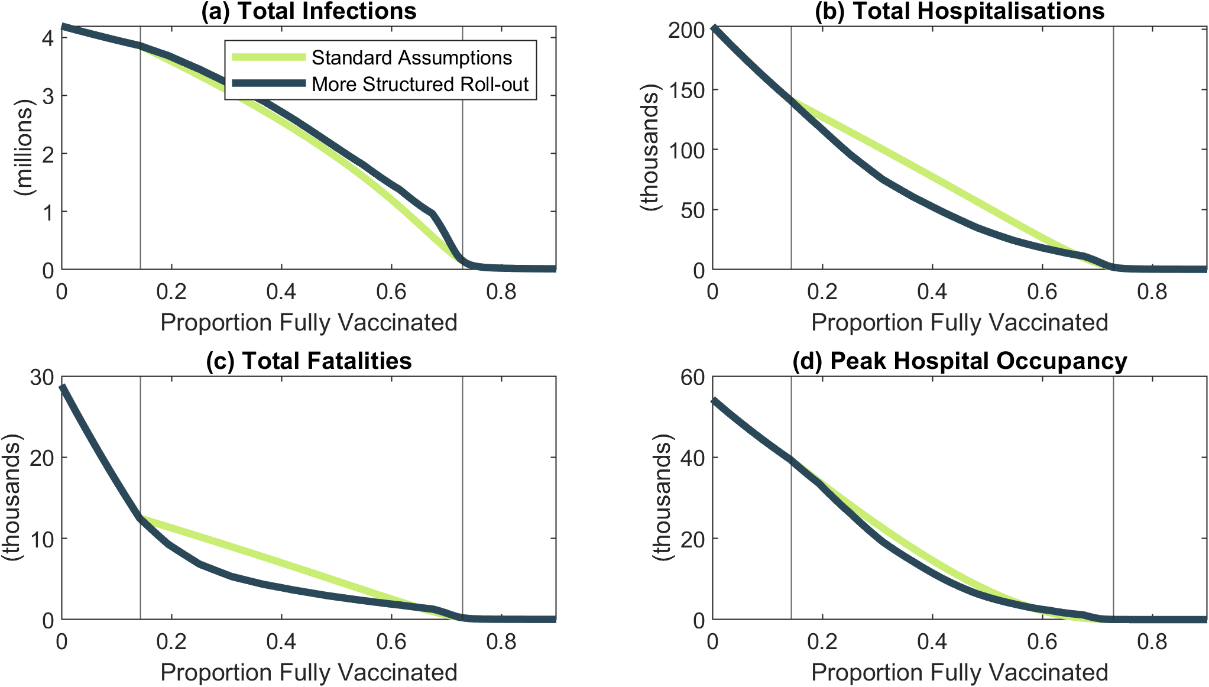


**Figure S7.** Total infections (a), hospitalisations (b), fatalities (c), and peak hospital occupancy (d) at the two vaccine rollouts described in the caption of Figure S6 and $R_{0}=3.0$. Results are from a 2-year simulation, assuming there is no further vaccination after the outbreak begins.


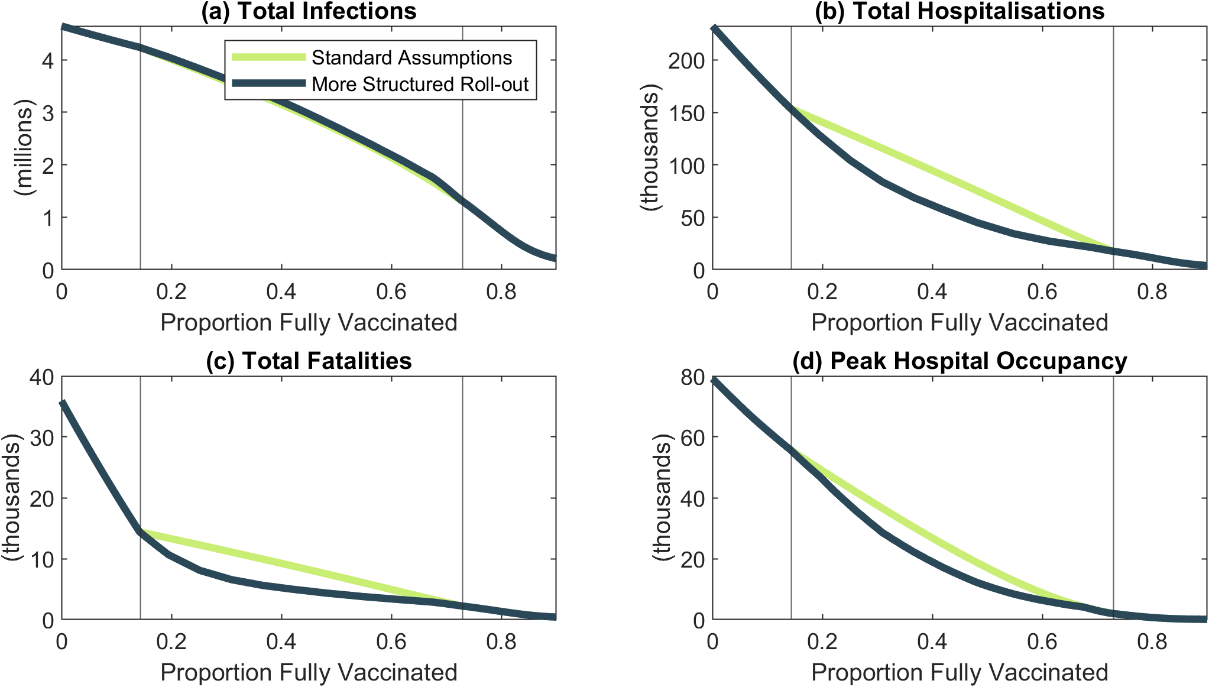


**Figure S8.** Total infections (a), hospitalisations (b), fatalities (c), and peak hospital occupancy (d) at the two vaccine rollouts described in the caption of Figure S6 and $R_{0}=4.5$. Results are from a 2-year simulation, assuming there is no further vaccination after the outbreak begins.


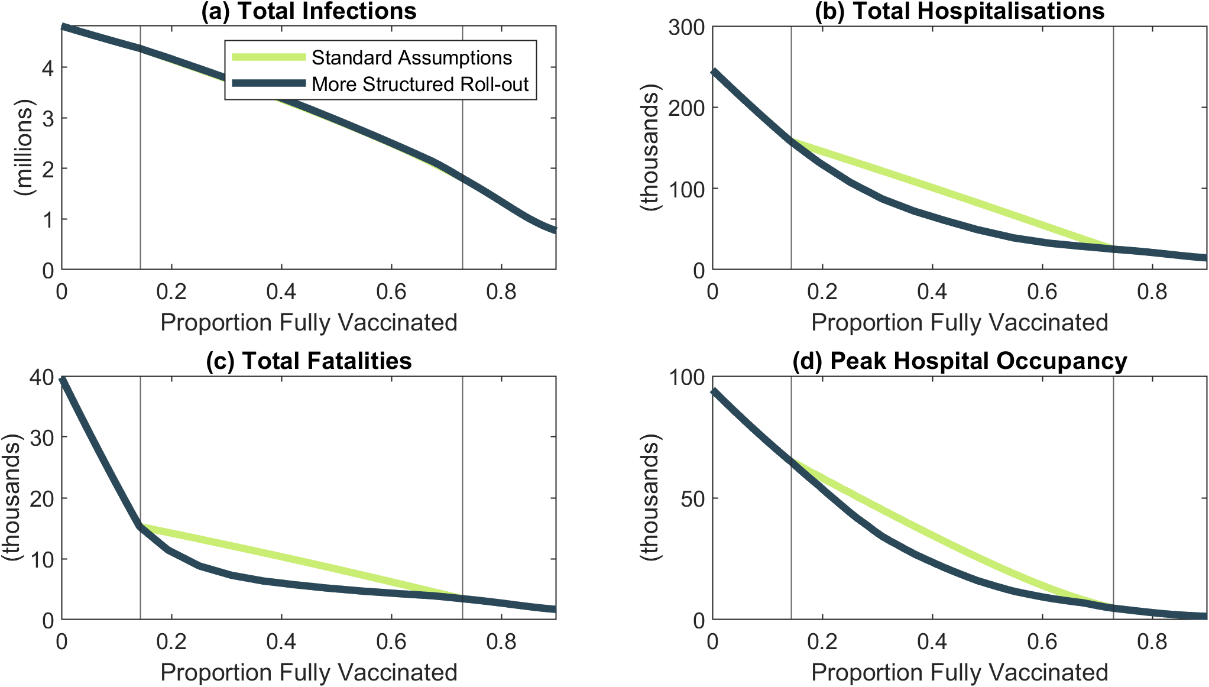


**Figure S9.** Total infections (a), hospitalisations (b), fatalities (c), and peak hospital occupancy (d) at the two vaccine rollouts described in the caption of Figure S6 and $R_{0}=6.0$. Results are from a 2-year simulation, assuming there is no further vaccination after the outbreak begins.

## 2.3 Sensitivity to vaccine infection-blocking assumptions

There are two ways in which a vaccine that prevents a proportion $e_{I}$ of infections is typically modelled. The first, often described as an “all-or-nothing” vaccine, is where a proportion $e_{I}$ of vaccinated individuals are completely immune to any infection and a proportion $1-e_{I}$ are completely susceptible. The second, often described as a “leaky” vaccine, assumes all vaccinated individuals have some likelihood of being infected given exposure, but that likelihood is reduced by a factor of $e_{I}$ relative to non-vaccinated individuals. There is, at present, limited evidence as to which of these assumptions is more realistic and it is possible that reality lies somewhere between the two (i.e. individuals are distributed along a spectrum of vaccine effectiveness). The results in the main text assume an “all-or-nothing” vaccine. Here, we investigate model outputs under a leaky vaccine assumption.

The choice of an all-or-nothing or a leaky vaccine assumption only affects how the accumulation of infection-acquired immunity changes the dynamics of an epidemic. The results on the effective reproduction number in the absence of any infection-acquired immunity (i.e. Figures 1-2 of the Main Text) are invariant to this assumption. The results from the stochastic implementation are also invariant to this assumption as infection-acquired immunity is ignored in this model. Previous modelling work also suggests that the choice of implementation has little effect on optimal vaccine roll-out strategies [3].

The choice of vaccine assumption only significantly affects the counterfactual scenarios where an epidemic wave occurs, ending due to the build-up of infection-acquired immunity in the population (e.g. Tables 3 and 4 and Figure 1 in the Main Text). Tables S4 and S5 reproduce the results from Tables 3 and 4 of the Main Text but with a leaky vaccine assumption. Vaccine effectiveness parameters are the same as in Table 1 of the Main Text.

| $\boldsymbol{R}_{\boldsymbol{0}}\boldsymbol{=4.5}$ | **Baseline** | **Lower effectiveness** | **Higher effectiveness** |
| --- | --- | --- | --- |
| $R_{v}$ | 1.47 | 1.86 | 1.35 |
| Infections | 1,800,000 | 3,300,00 | 760,000 |
| Hospitalisations | 24,000 | 43,000 | 9,700 |
| Fatalities | 3,000 | 5,600 | 1,200 |

**Table S4.** Results from a 2-year unmitigated epidemic with a leaky vaccine and 90% coverage of over 15-year-olds.

| $\boldsymbol{R}_{\boldsymbol{0}}\boldsymbol{=4.5}$ | **Baseline** | **Lower effectiveness** | **Higher effectiveness** |
| --- | --- | --- | --- |
| $R_{v}$ | 0.92 | 1.58 | 0.47 |
| Infections | 320,000 | 2,500,000 | 8,700 |
| Hospitalisations | 5,700 | 36,000 | 240 |
| Fatalities | 610 | 4,500 | 26 |

**Table S5.** Results from a 2-year unmitigated epidemic with a leaky vaccine and 90% coverage of the total population.

In scenarios where $R_{v}$ is high (e.g. low vaccine effectiveness, low vaccination coverage, or high $R_{0})$, the leaky vaccine assumption leads to substantially higher numbers of infections, hospitalisations, and deaths than the all-or-nothing vaccine assumption does. When $R_{v}$ is low the results are very similar [32].

## 2.4 Approval for 12+ year-olds only

Some countries have approved the Pfizer-BioNTech vaccine for use in children 12 years old and above. As an approximation, vaccinating 90% of 12-14-year-olds is similar to vaccinating 54% of 10-14-year-olds (90% of three out of five of the ages). We reproduce Table 3 from the Main Text with these additional vaccinations in Table S6.

When $R_{0}=3.0$, these additional vaccinations have a particularly large effect in the baseline vaccine effectiveness scenario. When only 15+ year-olds are vaccinated, $R_{v}$ is estimated to be 0.98, or fairly close to 1. The additional vaccines administered lower this to 0.82, significantly decreasing the number of infections from 150,000 to 34,000.

When $R_{0}=4.5$, these additional vaccinations are still insufficient to reach the population immunity threshold, although in the higher effectiveness scenario $R_{v}=1.03$ is fairly close (compared to $R_{v}=1.35$ when only 15+ year-olds are vaccinated). Minor social distancing and/or testing and tracing measures would be sufficient to bring $R_{eff}$ below 1 in this scenario.

| $\boldsymbol{R}_{\boldsymbol{0}}\boldsymbol{=3.0}$ | **Baseline** | **Lower effectiveness** | **Higher effectiveness** |
| --- | --- | --- | --- |
| Vaccine effectiveness | $e_{I}=70\%, e_{T}=50\%$ | $e_{I}=50\%, e_{T}=40\%$ | $e_{I}=90\%, e_{T}=50\%$ |
| $R_{v}$ | 0.82 | 1.16 | 0.69 |
| Infections | 34,000 (53%) | 880,000 (67%) | 9,500 (26%) |
| Hospitalisations | 500 (35%) | 12,000 (47%) | 160 (15%) |
| Fatalities | 56 (35%) | 1,400 (47%) | 18 (15%) |
| Peak in hospital | N/A | 630 (after 240 days) | N/A |
| $\boldsymbol{R}_{\boldsymbol{0}}\boldsymbol{=4.5}$ | **Baseline** | **Lower effectiveness** | **Higher effectiveness** |
| $R_{v}$ | 1.22 | 1.74 | 1.03 |
| Infections | 960,000 (50%) | 2,000,000 (64%) | 280,000 (22%) |
| Hospitalisations | 14,000 (35%) | 29,000 (47%) | 4,000 (15%) |
| Fatalities | 1,700 (35%) | 3,700 (47%) | 480 (15%) |
| Peak in hospital | 1,100 (after 180 days) | 4,500 (after 100 days) | 120 (after 310 days) |
| $\boldsymbol{R}_{\boldsymbol{0}}\boldsymbol{=6.0}$ | **Baseline** | **Lower effectiveness** | **Higher effectiveness** |
| $R_{v}$ | 1.63 | 2.32 | 1.38 |
| Infections | 1,600,000 (49%) | 2,500,000 (63%) | 800,000 (22%) |
| Hospitalisations | 23,000 (35%) | 36,000 (47%) | 12,000 (15%) |
| Fatalities | 3,000 (35%) | 5,100 (47%) | 1,500 (15%) |
| Peak in hospital | 3,500 (after 110 days) | 8,100 (after 70 days) | 1,300 (after 150 days) |

**Table S6.** Results from an unmitigated epidemic and 90% coverage of over 12-year-olds. This is modelled by assuming 54% coverage of 10-14-year-olds.

## 2.5 Deterministic SEIR sensitivity to other epidemiological parameters

We consider the sensitivity of various outputs of the deterministic SEIR implementation to the basic reproduction number (Figure S10), the rate of imported infections (Figure S11), the mean length of hospital stay (Figure S12), the mean latent period (Figure S13), and the mean infectious period (Figure S14).

## 2.6 Effect of testing and case isolation on reproduction number

Results in the main paper (Table 2) on the level of vaccine coverage required to reach the population immunity threshold assume there are no non-pharmaceutical interventions. Results from the stochastic implementation in the main paper assume that case isolation and contact tracing begin once a new outbreak is detected. This is reasonable under an elimination strategy coupled with strong border measures designed to keep COVID-19 out of the community. However, due to capacity constraints, the impact contact tracing system on transmission would be far smaller if there were regular imported cases triggering multiple outbreaks simultaneously.

An intermediate situation between: (i) no non-pharmaceutical interventions and (ii) intensive contact tracing for small, sporadic outbreaks is where some baseline case-targeted control measures remain in place. To investigate this, we calculated the model-implied reduction in effective reproduction number with: $p_{trace}=0$ (no contact tracing), $p_{detect}=70\%$ (significantly increased detection rates), and a reduced mean time from symptom onset to detection of 2 days. Assuming case isolation is 100% effective in preventing transmission, this reduces the reproduction number by an estimated 14%, as per Eq. (S4), compared to no control. If case isolation is imperfect and reduces transmission by 80%, this reduces the reproduction number by an estimated 11%. This is a much smaller reduction in $R_{eff}$ than can be achieved by intensive contact tracing of small outbreaks, but it does mean that effective population immunity ($R_{eff}<1$ with widespread testing and case isolation but without mass restrictions) can be achieved with a slightly lower vaccine coverage (Table S7).


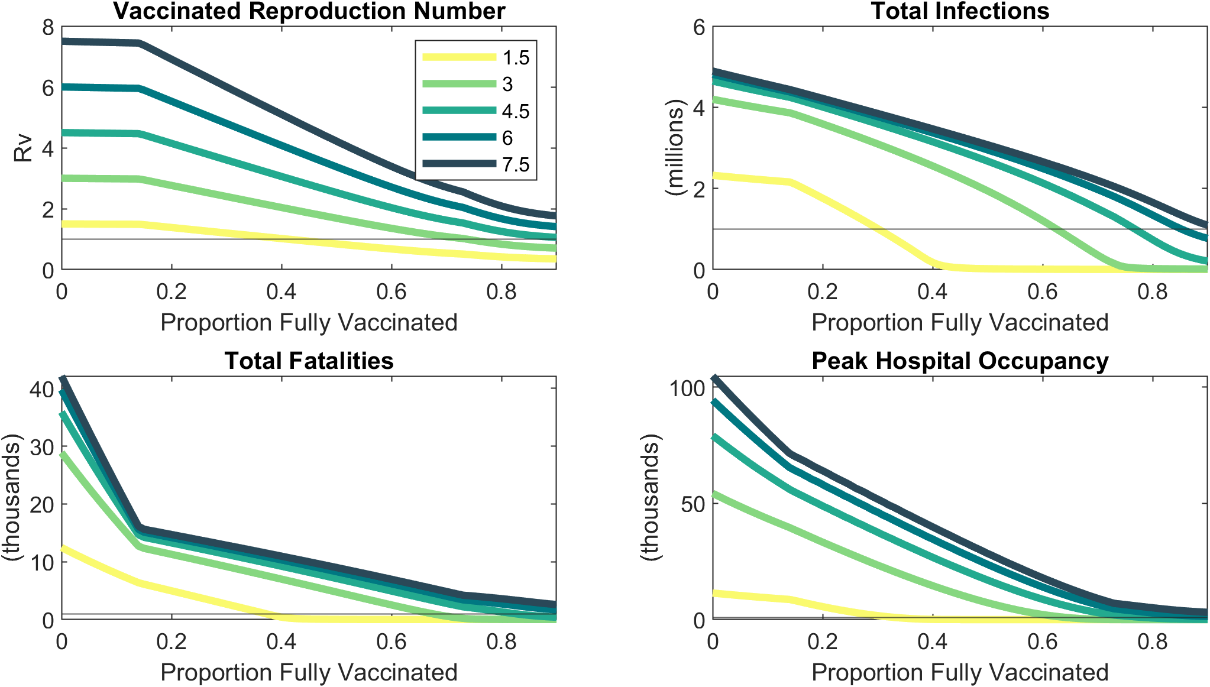


**Figure S10.** Sensitivity to $R_{0}$, testing values between 1.5 and 7.5 in increments of 1.5. When $R_{0}=1.5$ and 3.0, the population immunity threshold can be reached without vaccinating under 15-year-olds. When $R_{0}=4.5$ vaccination of under-15-year-olds is required. When $R_{0}=6.0$ and $7.5$ the population immunity threshold cannot be reached without greater than 90% population coverage. Furthermore, as $R_{0}$ increases, the number of infections, fatalities, and peak hospital occupancy also increase.


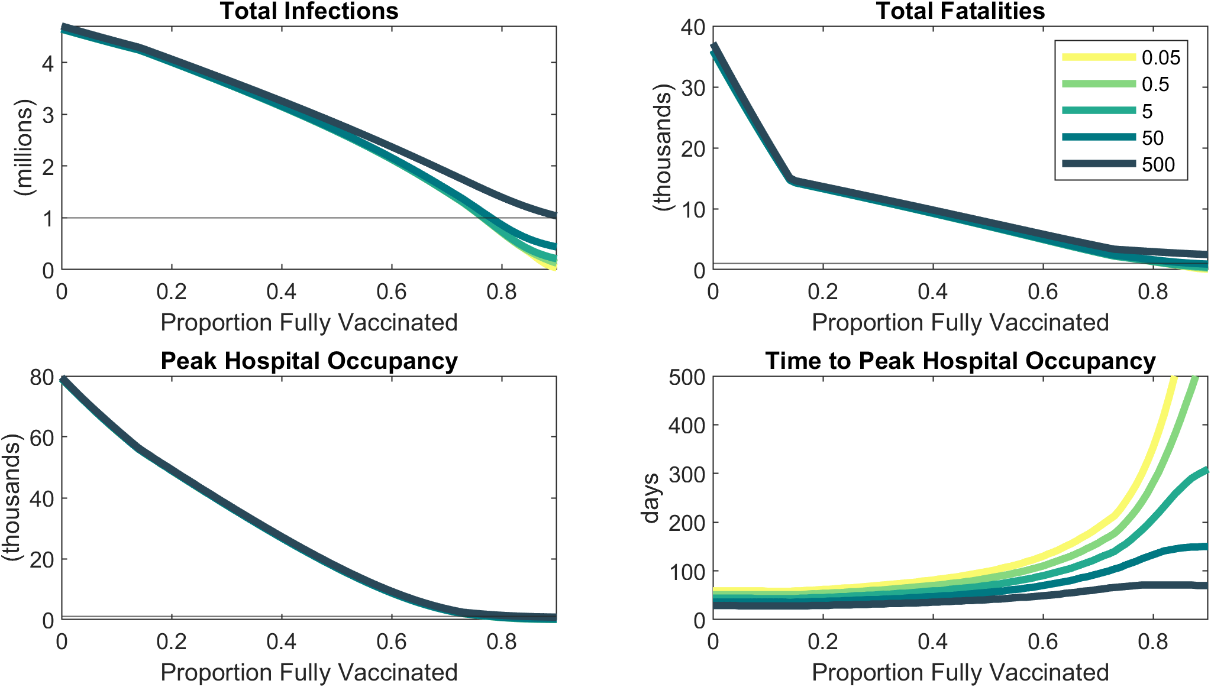


**Figure S11.** Sensitivity to the rate of imported cases (from 0.05 to 500 imported infectious cases per day). Until high levels of vaccination are reached ($R_{v}<1)$, differences in rates of imported cases makes little difference to the overall results, except in extremely high infected arrival rates (500 per day). The timing of the epidemic peak is more sensitive, with higher arrival rates resulting in an earlier peak. This scenario assumes $R_{0}=4.5$.


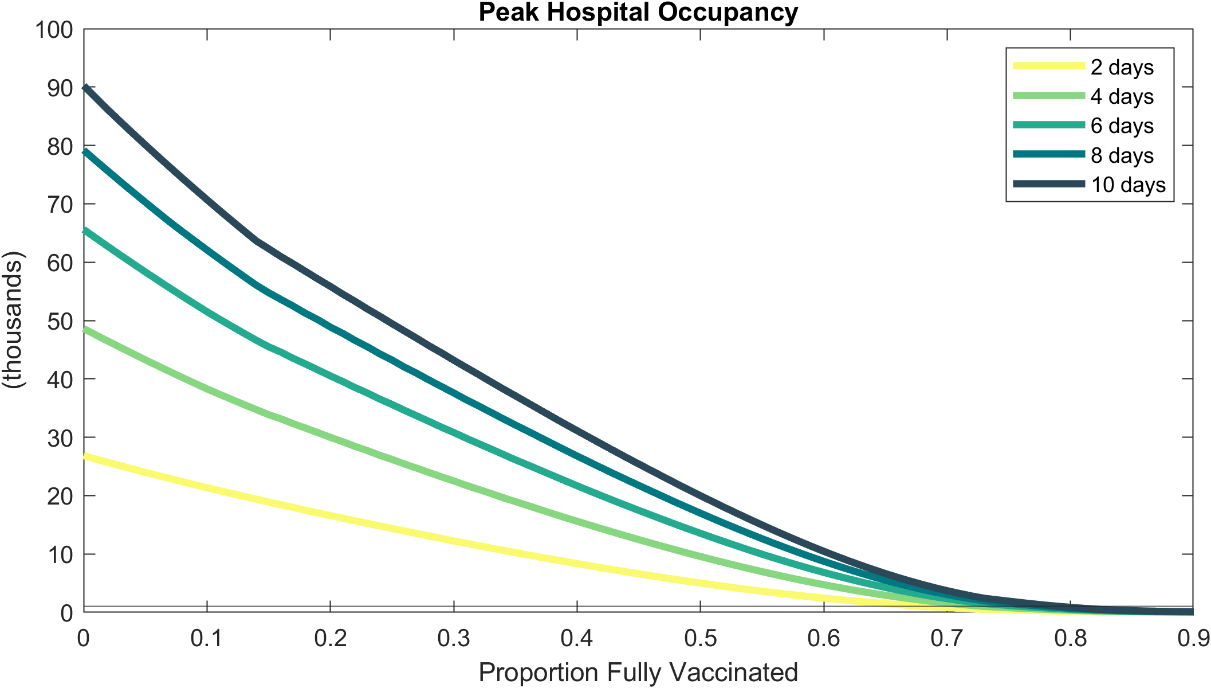


**Figure S12.** Sensitivity to the mean length of hospital stay, testing values between 2 and 10 days. This parameter only has implications on the peak hospital occupancy, with longer times in hospital resulting in a larger peak occupancy. In reality this likely also varies with vaccination coverage as different age groups have different expected hospitalisation durations, although this is not considered in our modelling. This scenario assumes $R_{0}=4.5$.


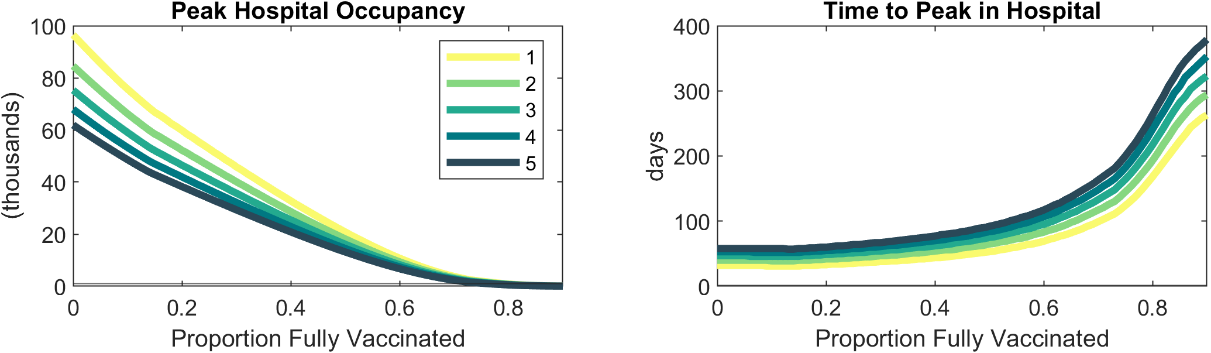


**Figure S13.** Sensitivity to the latent period (mean time $t_{E}$ in “exposed” compartment), testing values between 1 and 5 days. This parameter only affects the timing and size of the epidemic peak, with longer latent periods implying lower peak hospital occupancy and longer time to the peak. This scenario assumes $R_{0}=4.5$.


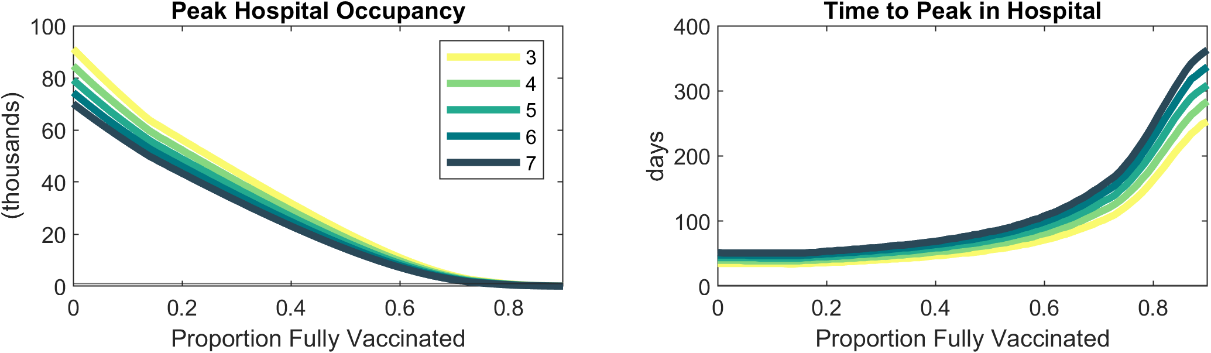


**Figure S14.** Sensitivity to the mean duration of the infectious period $t_{I}$ (for fixed $R_{0}=4.5$), testing values between 3 and 7 days. There is a similar effect in varying this as with the latent period.

|  | **Baseline** | **Lower effectiveness** | **Higher effectiveness** |
| --- | --- | --- | --- |
| Vaccine effectiveness | $e_{I}=70\%, e_{T}=50\%$ | $e_{I}=50\%, e_{T}=40\%$ | $e_{I}=90\%, e_{T}=50\%$ |
| $R_{0}=3.0$ | 68% | 88% | 62% |
| $R_{0}=4.5$ | 85% | - | 77% |
| $R_{0}=6.0$ | 96%* | - | 83% |

**Table S7.** Vaccine coverage required to achieve effective population immunity ($R_{eff}<1$) with case-targeted control such that 70% of cases are isolated an average of 2 days after symptom onset, with subsequent transmission reduced by 80%, providing a reduction in the effective reproduction number of 11%. Results are shown for each vaccine effectiveness scenario at three different values of $R_{0}$. Estimates assume a structured roll-out, beginning in 65+ year-olds, then 15-64 year-olds, and finally under 15-year olds, with up to 90% of each group vaccinated. Estimates with an asterisk are greater than 90% and assume equal coverage in all age groups.

## 2.7 Stochastic branching process sensitivity to vaccine effectiveness

Here we investigate the effect of the lower and higher vaccine effectiveness assumptions on results of the stochastic branching process (Table S8). Other parameters and assumptions are the same as in the “Control of border-related outbreaks” section of the main paper – these include contact tracing and case isolation amounting to a 44% reduction in the reproduction number. Vaccine coverage is assumed to be 90% of over 15-year-olds.

| $\boldsymbol{R}_{\boldsymbol{0}}\boldsymbol{=3.0}$ | **Baseline** | **Lower effectiveness** | **Higher effectiveness** |
| --- | --- | --- | --- |
| Vaccine effectiveness | $e_{I}=70\%, e_{T}=50\%$ | $e_{I}=50\%, e_{T}=40\%$ | $e_{I}=90\%, e_{T}=50\%$ |
| $R_{v}$ | 1.02 | 0.93 | 1.60 |
| Infections at detection | 6 (1, 58) | 12 (1, 77) | 3 (1, 45) |
| P(elim before 1000 infs) | 100% | 100% | 100% |
| Time to elimination | 12 (0, 26) | 15 (0, 31) | 10 (0, 23) |
| Total hospitalisations | 0 (0, 2) | 0 (0, 2) | 0 (0, 1) |
| $\boldsymbol{R}_{\boldsymbol{0}}\boldsymbol{=4.5}$ | **Baseline** | **Lower effectiveness** | **Higher effectiveness** |
| $R_{v}$ | 1.47 | 1.86 | 1.35 |
| Infections at detection | 14 (1, 97) | 22 (1, 142) | 8 (1, 83) |
| P(elim before 1000 infs) | 99.7% | 82.7% | 99.98% |
| Time to elimination | 17 (0, 34) | 22 (2, 42) | 13 (0, 32) |
| Total hospitalisations | 0 (0, 2) | 0 (0, 4) | 0 (0, 2) |
| $\boldsymbol{R}_{\boldsymbol{0}}\boldsymbol{=4.5}$ | **Baseline** | **Lower effectiveness** | **Higher effectiveness** |
| $R_{v}$ | 2.04 | 1.86 | 3.20 |
| Infections at detection | 22 (1, 154) | 34 (1, 217) | 15 (1, 126) |
| P(elim before 1000 infs) | 81.4% | 61.2% | 93.1% |
| Time to elimination | 22 (1, 44) | 29 (3, 56) | 18 (0, 40) |
| Total hospitalisations | 0 (0, ,4) | 1 (0, 6) | 0 (0, 3) |

**Table S8.** Branching process sensitivity to vaccine effectiveness against infection and transmission with 90% of 15+ year-olds vaccinated. Default values are used for all parameters (see Table S1). Median values from 10,000 trials reported with 95% confidence intervals in parenthesis. Probability of elimination before 1,000 infections assumes effective and scalable contact tracing and case isolation takes place after detection. Time to elimination and total hospitalisations assume population-level controls are used in addition to contact tracing and case isolation.

## 2.8 Stochastic branching process sensitivity to other epidemiological parameters

We also test sensitivity to $R_{0}$, $t_{detect}$, $p_{detect}^{pre}$, $t_{trace}$, and $p_{trace}$ in Tables S9, S10, S11, S12 and S13. Two stages of vaccination are now considered: no vaccination and 90% coverage of 15+ year-olds. Once an outbreak is detected, population level controls are implemented in addition to contact tracing and case isolation, which provides an overall 81% reduction in $R$ under default parameters. Reported values for infections at detection, time to elimination, and total hospitalisations are medians. P(elim) is the proportion of simulations that resulted in elimination before 1,000 infections, with the aforementioned contact tracing and case isolation operating.

| $R_{0}$ | No vaccination | | | | | 90% coverage of 15+ | | | | |
| --- | --- | --- | --- | --- | --- | --- | --- | --- | --- | --- |
|  | 1.5 | 2.5 | 3.5 | 4.5 | 5.5 | 1.5 | 2.5 | 3.5 | 4.5 | 5.5 |
| Infections at det | 13 | 38 | 66 | 102 | 138 | 2 | 7 | 15 | 21 | 31 |
| P(elim) (%) | 100 | 61 | 48 | 41 | 36 | 100 | 100 | 100 | 82 | 65 |
| Time to elimination | 17 | 33 | 70 | N/A | N/A | 8 | 12 | 17 | 21 | 27 |
| Total hosps | 1 | 3 | 16 | N/A | N/A | 0 | 0 | 0 | 0 | 1 |

**Table S9.** Branching process sensitivity to varying values of $R_{0}$. As $R_{0}$ increases the infections at detection, time to elimination, and total hospitalisations from a mitigated outbreak increase and probability of elimination decreases. In the no vaccination scenario with $R_{0}=5.5$, contact tracing and default population level controls are not sufficient to control an outbreak, with $R_{eff}>1$.

| $t_{detect}$ (days) | No vaccination | | | | | 90% coverage of 15+ | | | | |
| --- | --- | --- | --- | --- | --- | --- | --- | --- | --- | --- |
|  | 1 | 2 | 3 | 4 | 5 | 1 | 2 | 3 | 4 | 5 |
| Infections at det | 41 | 51 | 58 | 70 | 77 | 11 | 12 | 13 | 14 | 15 |
| P(elim) (%) | 49 | 49 | 49 | 49 | 47 | 100 | 100 | 100 | 100 | 99 |
| Time to elimination | 50 | 58 | 65 | 72 | 77 | 16 | 17 | 17 | 17 | 17 |
| Total hosps | 7 | 10 | 12 | 16 | 20 | 0 | 0 | 0 | 0 | 0 |

**Table S10.** Branching process sensitivity to varying values of mean delay from symptom onset to case detection. As the delay from onset to detection increases, the number of infections at detection increase substantially, particularly in the no vaccination scenario.

| $p_{detect}^{pre}$ | No vaccination | | | | | 90% coverage of 15+ | | | | |
| --- | --- | --- | --- | --- | --- | --- | --- | --- | --- | --- |
|  | 5% | 10% | 20% | 50% | 80% | 5% | 10% | 20% | 50% | 80% |
| Infections at det | 175 | 85 | 38 | 11 | 6 | 34 | 17 | 8 | 3 | 2 |
| P(elim) (%) | 47 | 47 | 49 | 52 | 56 | 99 | 99 | 100 | 100 | 100 |
| Time to elimination | 97 | 78 | 56 | 30 | 21 | 21 | 18 | 14 | 11 | 10 |
| Total hosps | 45 | 20 | 8 | 1 | 1 | 0 | 0 | 0 | 0 | 0 |

**Table S11.** Branching process sensitivity to varying values of probability of detecting a symptomatic case before an outbreak is detected. As probability of detection increases, the number of infections at detection increases, as does the probability of elimination. In scenarios where $p_{detect}^{pre}$ is greater than the default value of $p_{detect}^{post}=40\%$, we increase $p_{detect}^{post}$ to match.

| $t_{trace}$ (days) | No vaccination | | | | | 90% coverage of 15+ | | | | |
| --- | --- | --- | --- | --- | --- | --- | --- | --- | --- | --- |
|  | 1 | 3 | 5 | 7 | 9 | 1 | 3 | 5 | 7 | 9 |
| P(elim) (%) | 52 | 49 | 48 | 48 | 48 | 100 | 100 | 100 | 99 | 96 |
| Time to elimination | 35 | 44 | 61 | 85 | 141 | 16 | 17 | 17 | 17 | 17 |
| Total hosps | 6 | 8 | 13 | 20 | 45 | 0 | 0 | 0 | 0 | 0 |

**Table S12.** Branching process sensitivity to varying values of mean delay from exposure to detection via contact tracing. As the delay in tracing increases, the probability of elimination before 1000 cases decreases.

| $p_{trace}$ | No vaccination | | | | | 90% coverage of 15+ | | | | |
| --- | --- | --- | --- | --- | --- | --- | --- | --- | --- | --- |
|  | 50% | 60% | 70% | 80% | 90% | 50% | 60% | 70% | 80% | 90% |
| P(elim) (%) | 48 | 48 | 48 | 49 | 48 | 95 | 98 | 100 | 100 | 100 |
| Time to elimination | N/A | 98 | 72 | 57 | 48 | 18 | 17 | 17 | 17 | 17 |
| Total hosps | N/A | 24 | 16 | 12 | 10 | 0 | 0 | 0 | 0 | 0 |

**Table S13.** Branching process sensitivity to varying values of probability of detecting an infected individual by contact tracing. As this increases, the probability of elimination increases.

# 3. Effectiveness of the Pfizer-BioNTech vaccine

In March 2021, the New Zealand government confirmed the purchase of sufficient doses of the Pfizer-BioNTech BNT162b2 mRNA vaccine to vaccinate the entire population. In this section, we first briefly justify our assumptions for vaccine effectiveness parameters. In the subsections below, we summarise published results on the efficacy and effectiveness of the Pfizer vaccine in the context of our model.

Lipsitch and Kahn [15] argue that vaccine efficacy against viral RT-PCR positivity is a plausible lower bound on the vaccine’s efficacy against transmission. The studies discussed in the “effectiveness against infection” subsection below are all variations on effectiveness against viral positivity studies, suggesting a lower bound on the overall reduction of transmission of around 90%. However, early results suggest there may be decreased effectiveness against variants of concern [14,33], and evidence is limited for the effect in young and old people, so we use $e_{I}=70\%$ effectiveness against infection and $e_{T}=50\%$ effectiveness against onwards transmission conditional on breakthrough infection. This gives an overall implied transmission reduction from vaccination of $1-\left( 1-e_{I} \right)\left( 1-e_{T} \right)=85\%$ and is in-line with modelling from the UK [17].

The effectiveness against symptomatic disease appears to be as high as 95% across many studies. We use 80% as our baseline estimate for $e_{D}$, which implies an overall 94% effectiveness against severe disease. Although more recent evidence has shown decreased effectiveness of 88% [85.3%, 90.1%] against symptomatic disease causes by the Delta variant [34], effectiveness against severe disease or death caused by either Alpha or Delta is estimated to be 90-99% [35,36].

## 3.1 Effectiveness against SARS-CoV-2 infection

Weekes, et al. [37] present evidence from healthcare workers in the UK for effectiveness against asymptomatic viral positivity. Data was analysed over two weeks from 18^th^ to 31^st^ January 2021 and included 4,408 PCR test-results in the first week and 4,411 in the second. The results found that 26/3,252 (0.8%) of tests from non-vaccinated HCWs were positive, 13/3,535 (0.37%) of tests from those <12 days post-vaccination were positive, and 4/1,989 (0.20%) of tests from those >12 days post-vaccination were positive. This suggests an approximate 75% effectiveness against viral positivity following a single-dose. Similar results were found when symptomatic individuals were included. All results were for a single-dose. This may be evidence of decreased infection duration or decreased overall susceptibility or a combination of both. In any case, the vaccine effectiveness against viral positivity (or equivalently, documented infection), is a plausible lower bound on effectiveness against transmission [15].

Dagan, et al. [10] in Israel found an effectiveness against documented infection of 46% (40%, 51%) between days 14 and 20 following the first dose, and 92% (88%, 95%) more than 7 days after the second. This is more pessimistic than [37] for single-dose effectiveness, but more optimistic long-term. This study is also based on a much larger sample size (>1m participants).

Chodick, et al. [11], also in Israel, found an effectiveness against documented infection of 51.4% in days 13-24 following the first dose. This was estimated by comparing individuals >13 days after their first dose with those after 1-12 days. As such, we place less weighting on this study.

Moustsen-Helms, et al. [13], in Denmark, found an effectiveness against viral positivity of 64% (14%, 84%) in long-term care facility residents and 90% (92%, 95%) in healthcare workers. There are many caveats, but these results may suggest that reduced effectiveness in older individuals is plausible.

Thompson, et al. [12], in the US, found an effectiveness against infection of 80% (59%, 90%) >= 14 days after the first dose, and an effectiveness against infection of 90% (68%, 97%) >= 14 days after the second dose.

Abu-Raddad, et al. [14], in Qatar, found an effectiveness against viral positivity for B.1.1.7 of 89.5% (85.9%, 92.3%) and an effectiveness against viral positivity for B.1.351 of 75.0% (70.5%, 78.9%) (both > 14 days after second dose). This is strong evidence for reduced effectiveness against B.1.351.

## 3.2 Effectiveness against transmission given breakthrough infection

Harris, et al. [16], in the UK, provide early evidence for additional prevention against onward transmission given breakthrough infection. The adjusted odds ratio for onward transmission conditional on being vaccinated with BNT162b2 was 0.51 (0.44, 0.59). Individuals in this study were considered vaccinated if they received their first dose at least 21 days prior to testing positive.

## 3.3 Effectiveness against symptomatic COVID-19

Polack, et al. [18] present the stage 2/3 clinical trial results. The primary endpoint was COVID-19 disease, to which the vaccine efficacy was found to be 95.0% (90.3%, 97.6%).

Dagan, et al. [10] in Israel found an effectiveness against symptomatic COVID-19 of 57% (50%, 63%) between days 14 and 20 after the first dose, and 94% (87%, 98%) more than 7 days after the second.

## 3.4 Effectiveness against severe disease/hospitalisation

Dagan, et al. [10] in Israel found an effectiveness against hospitalisation of 74% (56%, 86%) between 14 and 20 days after the first dose, and 87% (55%, 100%) at least a week after the second. Similarly, they found an effectiveness against severe disease of 62% (39%, 80%) and 92% (75%, 100%).

Abu-Raddad, et al. [14] found effectiveness against severe (or worse) disease of 100% for both B.1.1.7 and B.1.351 with confidence intervals of (81.7%, 100%) and (73.7%, 100%) respectively. Against all SARS-CoV-2 they found an effectiveness of 97.4% (99.2%, 99.5%).

## 3.5 Effectiveness against death

Dagan, et al. [10] in Israel found an effectiveness against death of 84% (44%, 100%) in individuals 21 to 27 days after their first dose. There was insufficient data to estimate this for individuals with multiple doses.

**References**

1 Grout, L. *et al.* Estimating the Failure Risk of Quarantine Systems for Preventing COVID-19 Outbreaks in Australia and New Zealand. *medRxiv preprint*, doi:10.1101/2021.02.17.21251946 (2021).

2 Ebaseh-Onofa, B. O. & Matis, J. H. Compartmental models with Erlang distributed residence times and random rate coefficients. *Bulletin of Mathematical Biology*. **54**, 929-938, doi:10.1007/bf02460659 (1992).

3 Bubar, K. M. *et al.* Model-informed COVID-19 vaccine prioritization strategies by age and serostatus. *Science*. **371**, 916-921, doi:10.1126/science.abe6959 (2021).

4 Verity, R. *et al.* Estimates of the severity of coronavirus disease 2019: a model-based analysis. *The Lancet Infectious Diseases*. **20**, 669-677, doi:10.1016/s1473-3099(20)30243-7 (2020).

5 Hinch, R. *et al.* OpenABM-Covid19-An agent-based model for non-pharmaceutical interventions against COVID-19 including contact tracing. *PLoS Comput Biol*. **17**, e1009146, doi:10.1371/journal.pcbi.1009146 (2021).

6 SAGE. Children’s Task and Finish Group: update to 4 November 2020 paper on children, schools and transmission - 17 December 2020, <<https://www.gov.uk/government/publications/tfc-children-and-transmission-update-paper-17-december-2020>> (2020).

7 Davies, N. G. *et al.* Age-dependent effects in the transmission and control of COVID-19 epidemics. *Nat Med*. **26**, 1205-1211, doi:10.1038/s41591-020-0962-9 (2020).

8 Dattner, I. *et al.* The role of children in the spread of COVID-19: Using household data from Bnei Brak, Israel, to estimate the relative susceptibility and infectivity of children. *PLoS Comput Biol*. **17**, e1008559, doi:10.1371/journal.pcbi.1008559 (2021).

9 Li, F. *et al.* Household transmission of SARS-CoV-2 and risk factors for susceptibility and infectivity in Wuhan: a retrospective observational study. *The Lancet Infectious Diseases*. **21**, 617-628, doi:10.1016/s1473-3099(20)30981-6 (2021).

10 Dagan, N. *et al.* BNT162b2 mRNA Covid-19 Vaccine in a Nationwide Mass Vaccination Setting. *N Engl J Med*. **384**, 1412-1423, doi:10.1056/NEJMoa2101765 (2021).

11 Chodick, G. *et al.* Assessment of Effectiveness of 1 Dose of BNT162b2 Vaccine for SARS-CoV-2 Infection 13 to 24 Days After Immunization. *JAMA Netw Open*. **4**, e2115985, doi:10.1001/jamanetworkopen.2021.15985 (2021).

12 Thompson, M. G. *et al.* Interim Estimates of Vaccine Effectiveness of BNT162b2 and mRNA-1273 COVID-19 Vaccines in Preventing SARS-CoV-2 Infection Among Health Care Personnel, First Responders, and Other Essential and Frontline Workers — Eight U.S. Locations, December 2020–March 2021. *MMWR. Morbidity and Mortality Weekly Report*. **70**, doi:10.15585/mmwr.mm7013e3 (2021).

13 Moustsen-Helms, I. R. *et al.* Vaccine effectiveness after 1st and 2nd dose of the BNT162b2 mRNA Covid-19 vaccine in long-term care facility residents and healthcare workers – a Danish cohort study. *medRxiv preprint*, doi:10.1101/2021.03.08.21252200 (2021).

14 Abu-Raddad, L. J., Chemaitelly, H., Butt, A. A. & National Study Group for, C.-V. Effectiveness of the BNT162b2 Covid-19 Vaccine against the B.1.1.7 and B.1.351 Variants. *N Engl J Med*. **385**, 187-189, doi:10.1056/NEJMc2104974 (2021).

15 Lipsitch, M. & Kahn, R. Interpreting vaccine efficacy trial results for infection and transmission. *Vaccine*. **39**, 4082-4088, doi:10.1101/2021.02.25.21252415 (2021).

16 Harris, R. J. *et al.* Effect of Vaccination on Household Transmission of SARS-CoV-2 in England. *N Engl J Med*. **385**, 759-760, doi:10.1056/NEJMc2107717 (2021).

17 Scientific Pandemic Influenza Group on Modelling. SPI-M-O: Summary of further modelling of easing restrictions – Roadmap Step 4, <<https://assets.publishing.service.gov.uk/government/uploads/system/uploads/attachment_data/file/993510/S1287_SPI-M-O_Summary_Roadmap_step_4.pdf>> (2021).

18 Polack, F. P. *et al.* Safety and Efficacy of the BNT162b2 mRNA Covid-19 Vaccine. *N Engl J Med*. **383**, 2603-2615, doi:10.1056/NEJMoa2034577 (2020).

19 Madhi, S. A. *et al.* Efficacy of the ChAdOx1 nCoV-19 Covid-19 Vaccine against the B.1.351 Variant. *N Engl J Med*. **384**, 1885-1898, doi:10.1056/NEJMoa2102214 (2021).

20 Ferretti, L. *et al.* Quantifying SARS-CoV-2 transmission suggests epidemic control with digital contact tracing. *Science*. **368**, doi:10.1126/science.abb6936 (2020).

21 Steyn, N. *et al.* Māori and Pacific People in New Zealand have higher risk of hospitalisation for COVID-19. *New Zealand Medical Journal*. **134**, 28-43 (2021).

22 James, A. *et al.* Successful contact tracing systems for COVID-19 rely on effective quarantine and isolation. *PLoS One*. **16**, e0252499, doi:10.1371/journal.pone.0252499 (2021).

23 StatsNZ. Infoshare: Estimated Resident Population by Age and Sex (Annual-Dec 2020), <<http://infoshare.stats.govt.nz>> (2020).

24 Chen, P. Z. *et al.* Heterogeneity in transmissibility and shedding SARS-CoV-2 via droplets and aerosols. *Elife*. **10**, doi:10.7554/eLife.65774 (2021).

25 James, A. *et al.* Model-free estimation of COVID-19 transmission dynamics from a complete outbreak. *PLoS One*. **16**, e0238800, doi:10.1371/journal.pone.0238800 (2021).

26 Adam, D. C. *et al.* Clustering and superspreading potential of SARS-CoV-2 infections in Hong Kong. *Nat Med*. **26**, 1714-1719, doi:10.1038/s41591-020-1092-0 (2020).

27 Prem, K., Cook, A. R. & Jit, M. Projecting social contact matrices in 152 countries using contact surveys and demographic data. *PLoS Comput Biol*. **13**, e1005697, doi:10.1371/journal.pcbi.1005697 (2017).

28 Mossong, J. *et al.* Social contacts and mixing patterns relevant to the spread of infectious diseases. *PLoS Med*. **5**, e74, doi:10.1371/journal.pmed.0050074 (2008).

29 Moore, S., Hill, E. M., Tildesley, M. J., Dyson, L. & Keeling, M. J. Vaccination and non-pharmaceutical interventions for COVID-19: a mathematical modelling study. *Lancet Infect Dis*. **21**, 793-802, doi:10.1016/S1473-3099(21)00143-2 (2021).

30 Prem, K. *et al.* Projecting contact matrices in 177 geographical regions: An update and comparison with empirical data for the COVID-19 era. *PLoS Comput Biol*. **17**, e1009098, doi:10.1371/journal.pcbi.1009098 (2021).

31 Nguyen, T. *et al.* COVID-19 vaccine strategies for Aotearoa New Zealand: a mathematical modelling study. *Lancet Reg Health West Pac*. **15**, 100256, doi:10.1016/j.lanwpc.2021.100256 (2021).

32 Ragonnet, R. *et al.* Vaccination Programs for Endemic Infections: Modelling Real versus Apparent Impacts of Vaccine and Infection Characteristics. *Sci Rep*. **5**, 15468, doi:10.1038/srep15468 (2015).

33 Liu, Y. *et al.* Neutralizing Activity of BNT162b2-Elicited Serum. *N Engl J Med*. **384**, 1466-1468, doi:10.1056/NEJMc2102017 (2021).

34 Lopez Bernal, J. *et al.* Effectiveness of Covid-19 Vaccines against the B.1.617.2 (Delta) Variant. *N Engl J Med*. **385**, 585-594, doi:10.1056/NEJMoa2108891 (2021).

35 Scientific Advisory Group for Emergencies. VEEP: Vaccine effectiveness table, 27 August 2021, <<https://www.gov.uk/government/publications/veep-vaccine-effectiveness-table-7-september-2021>> (2021).

36 UK Health Security Agency. COVID-19 vaccine surveillance report (week 49), <<https://www.gov.uk/government/publications/covid-19-vaccine-weekly-surveillance-reports>> (2021).

37 Weekes, M. *et al.* Single-dose BNT162b2 vaccine protects against asymptomatic SARS-CoV-2 infection. *Authorea Preprint*, doi:10.22541/au.161420511.12987747/v1 (2021).
